# Spatially segregated multiomics decodes metformin-mediated function-specific metabolic characteristics in diabetic kidney disease

**DOI:** 10.1093/lifemeta/loaf019

**Published:** 2025-05-30

**Authors:** Shi Qiu, Dandan Xie, Sifan Guo, Zhibo Wang, Xian Wang, Ying Cai, Chunsheng Lin, Hong Yao, Yu Guan, Qiqi Zhao, Qiang Yang, Yiqiang Xie, Songqi Tang, Aihua Zhang

**Affiliations:** International Advanced Functional Omics Platform, Scientific Experiment Center, Public Research Center, Hainan Medical University, Haikou, Hainan 571199, China; International Advanced Functional Omics Platform, Scientific Experiment Center, Public Research Center, Hainan Medical University, Haikou, Hainan 571199, China; International Advanced Functional Omics Platform, Scientific Experiment Center, Public Research Center, Hainan Medical University, Haikou, Hainan 571199, China; International Advanced Functional Omics Platform, Scientific Experiment Center, Public Research Center, Hainan Medical University, Haikou, Hainan 571199, China; International Advanced Functional Omics Platform, Scientific Experiment Center, Public Research Center, Hainan Medical University, Haikou, Hainan 571199, China; International Advanced Functional Omics Platform, Scientific Experiment Center, Public Research Center, Hainan Medical University, Haikou, Hainan 571199, China; Graduate School, Second Affiliated Hospital, Heilongjiang University of Chinese Medicine, Harbin, Heilongjiang 150040, China; Graduate School, Second Affiliated Hospital, Heilongjiang University of Chinese Medicine, Harbin, Heilongjiang 150040, China; First Affiliated Hospital, Harbin Medical University, Harbin, Heilongjiang 150040, China; Graduate School, Second Affiliated Hospital, Heilongjiang University of Chinese Medicine, Harbin, Heilongjiang 150040, China; Graduate School, Second Affiliated Hospital, Heilongjiang University of Chinese Medicine, Harbin, Heilongjiang 150040, China; Graduate School, Second Affiliated Hospital, Heilongjiang University of Chinese Medicine, Harbin, Heilongjiang 150040, China; International Advanced Functional Omics Platform, Scientific Experiment Center, Public Research Center, Hainan Medical University, Haikou, Hainan 571199, China; International Advanced Functional Omics Platform, Scientific Experiment Center, Public Research Center, Hainan Medical University, Haikou, Hainan 571199, China; International Advanced Functional Omics Platform, Scientific Experiment Center, Public Research Center, Hainan Medical University, Haikou, Hainan 571199, China; Graduate School, Second Affiliated Hospital, Heilongjiang University of Chinese Medicine, Harbin, Heilongjiang 150040, China

**Keywords:** spatial metabolomics, spatial proteomics, anatomic multiregion, metabolism, target

## Abstract

Understanding the specific metabolic changes in multiple regions of the kidney is crucial to revealing the underlying mechanism and developing effective targets for diabetic nephropathy (DN). In this study, integrated spatially resolved metabolomics and proteomics combined with mass spectrometry imaging (MSI) revealed a multi-scale region profile of the diabetic kidney. Based on anatomic location, spatial metabolomics revealed eight region-specific metabolite biomarkers uniquely localized to kidney segments, which were closely correlated to the clinical parameters of patients with DN. Specifically, treatment with metformin (MET) enriched inosinic acid, adenosine 3′,5′-diphosphate, nicotinamide adenine dinucleotide (NADH), and hydrated NADH (NADHX) levels in the cortex (Cor) and the outer stripe of kidney medulla (OM) anatomical subregions, while in the inner stripe of kidney medulla (IM) segmentation, the p-cresol sulfate level was downregulated. Comparing differently expressed proteins in each region showed that nephrosis 2 (Nphs2) was the highest loading feature. A further region-specific analysis of pathway enrichment characteristics indicated that the purine and ether lipid metabolism pathways were enriched in the Cor and OM regions, whereas the pantothenate and coenzyme A (CoA) biosynthesis pathways were mainly enriched in the IM region in response to MET treatment. Taken together, the spatially segregated metabolomics and proteomics studies reveal MET-mediated proteins and function-specific therapeutic pathways related to the anatomical multiregion of diabetic mouse kidneys.

## Introduction

Diabetic nephropathy (DN) is one of the most common complications of diabetes and a leading cause of end-stage renal disease. The number of DN patients has been increasing annually, posing an enormous burden on global public health [1–3]. The complete pathogenesis of DN is still lacking, and current treatment cannot completely prevent its progression. Therefore, there is an urgent need to further explore the pathogenesis of DN and uncover new therapeutic targets. Recent evidence suggests that metabolism plays a key role in the pathogenesis of DN [4–6]. Metformin (MET) is the most commonly used drug to treat type 2 diabetes. In addition to regulating blood glucose levels, it protects renal tubular cells as an effective antioxidant and alleviates DN by protecting podocytes [7, 8]. MET has been shown to regulate many metabolic pathways, thereby slowing down or preventing the progression of DN [9, 10]. DN is characterized by a progressive and chronic loss of kidney function. The kidney, as a highly metabolic organ, plays a key role in waste clearance, fluid balance, blood pressure regulation, and various endocrine functions. Anatomically speaking, two main kidney regions, namely the cortical region that produces filtrate and the medullary region where urine is concentrated, are responsible for different biological functions.

Due to the complexity and heterogeneity of kidney structure and function, the mechanism of DN development has not been fully elucidated. The cortex (Cor) and medulla are the two main anatomical structures of the kidney. Numerous studies have previously confirmed a close relationship between kidney disease and metabolic abnormalities [11–13]. Therefore, understanding the metabolic characteristics of these different anatomical regions is crucial for identifying new therapeutic targets. However, the complexity of kidney tissue structure and physiological functions makes it extremely difficult to reach a comprehensive evaluation for DN treatment. Although spatial background is often crucial for understanding biological processes, interpreting subregion-specific metabolism and protein profiles is essential for exerting tissue-specific functions and maintaining homeostasis. However, there is still limited research on kidney regions in spatial genomics, which loses spatial background. More importantly, the spatial organization and the function of the kidney are fundamentally linked. Therefore, it is important to systematically explore biomarkers in mouse kidney anatomical regions and subregions in a high-throughput manner.

Metabolism is an important aspect of biological systems that should be considered in understanding tissue homeostasis and pathogenesis [14, 15]. Recently, mass spectrometry imaging (MSI) has been able to characterize the spatial distribution of hundreds of molecules in tissue samples and explore the relationship between phenotype and metabolic characteristics, while preserving the spatial background of the tissue [16–18]. By revealing the spatial characteristics of different molecular spectra, spatial metabolomics can provide valuable insights into various fields of biology. MSI utilizes spatial virtual segmentation to delineate multiple regions of interest and visualizes the identification of these regions of interest to locate molecules, which is crucial for deciphering the potential mechanisms of biological phenomena in complex systems [19]. Spatial proteomics has unique advantages as it can specifically evaluate protein regulation in diseases [20]. Integrating proteomics with metabolomics can provide a deeper understanding of the spatial relationship between metabolism and proteins.

Understanding the precise location of analytes in kidney tissues may provide information about organ function. MET exerts therapeutic effects on DN through multi-target mechanisms [21]. It can significantly improve renal function markers and preserve histological structure, highlighting its multi-dimensional renoprotective efficacy. While MET’s systemic metabolic effects are known, its spatially compartmentalized actions in kidney microregions remain uncharacterized due to technical limitations in conventional omics. The integration of spatial metabolomics and proteomics data can reveal the complex relationships between proteins and metabolites in the pathogenesis of DN. The revolutionary technology of kidney tissue imaging and multiomics could construct three-dimensional tissue maps with unprecedented spatial and high-dimensional molecular resolution, providing a way to achieve precise treatment of kidney diseases. In this study, we have conducted spatial metabolomics analysis on the kidney tissue of the DN mouse model using MSI technology, and explored the spatial distribution characteristics of the differential metabolite and protein expression patterns, providing detailed information on the molecular pathological features of mouse kidney tissue.

## Results

### Spatial metabolomic characterization of diabetic mouse kidney

**Figure 1:**
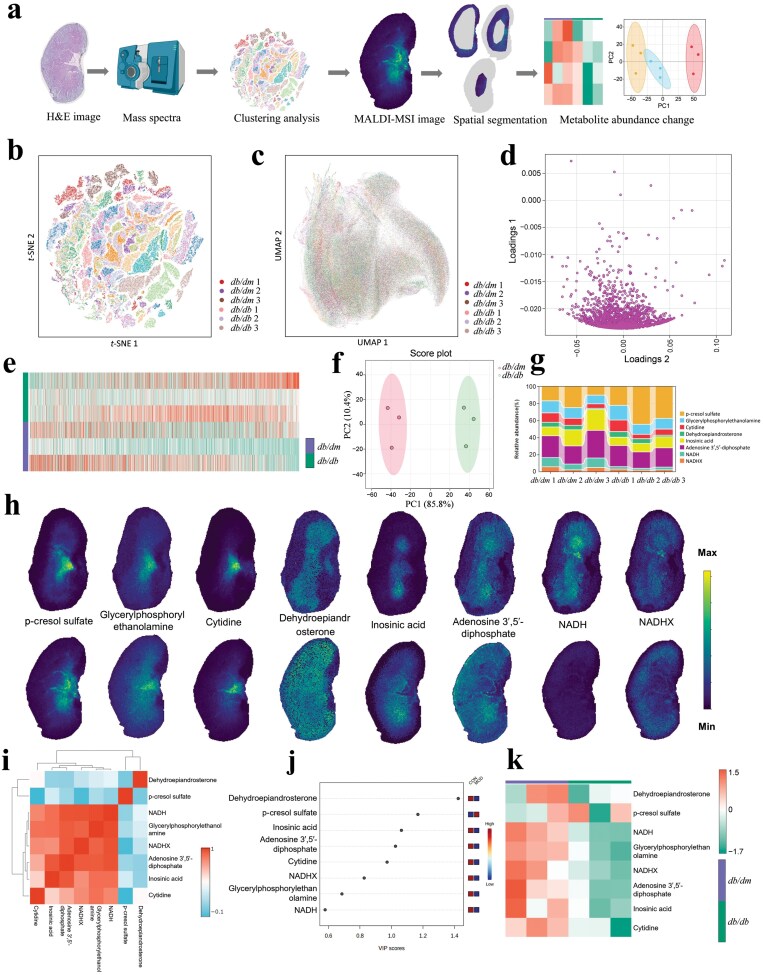
Spatial metabolomic characterization of diabetic mouse kidney. (a) The workflow of the spatial metabolomic platform includes slicing, mass spectra acquired by MSI, clustering analysis, MALDI-MSI image and spatial segmentation, and metabolite abundance change. (b) *t*-SNE plot displaying metabolite clusters. (c) UMAP plot visualizing the spatial distribution of metabolites in *db/db* mouse kidney tissue. (d) PLS loading plot of metabolites in *db/db* mouse kidney tissue. (e) Heatmap plot of metabolites in comparisons of *db/dm* and transgenic *db/db* mice. (f) PCA of variation between kidney samples of *db/dm* and transgenic *db/db* mice. (g) Dynamic stacking connection diagram showing relative intensity levels of metabolites between the *db/dm* and transgenic *db/db* mice. (h) MALDI-MSI analysis of mouse kidney biomarkers and their corresponding spatial distribution in kidney tissue sections. (i) Pearson correlation analyses of metabolite biomarkers in *db/db* mouse kidney tissue. (j) VIP scores with the top metabolites in diabetic mouse kidney samples. The X-axis represents the VIP score, and the Y-axis represents the metabolites. (k) Heatmap of biomarkers showing abundance differences between *db/dm* and *db/db* mice.

The workflow of the spatial metabolomics platform is shown in Fig. 1a, including slicing, mass spectra, clustering analysis, matrix-assisted laser desorption/ionization mass spectrometry imaging (MALDI-MSI) image, spatial segmentation, and metabolite abundance change. The well-defined mouse kidney regions clustered together according to the expression pattern using *t*-distributed stochastic neighbor embedding (*t*-SNE) (Fig. 1b) and Uniform Manifold Approximation and Projection (UMAP) analysis (Fig. 1c). Next, we performed partial least squares (PLS) analysis of metabolite level in both groups and obtained differential metabolites as shown in the loading plot (Fig. 1d). Heatmap showed that metabolite abundance differed in *db/dm* and transgenic *db/db* mice (Fig. 1e). Principal component analysis (PCA) suggested that *db/dm* and transgenic *db/db* mice were significantly different (Fig. 1f). The metabolite biomarkers in kidney tissues of *db/db* mice were selected from Z-score analysis (Supplementary Fig. S1; Supplementary Table S1). The dynamic stacking connection diagram showed the relative intensity levels of metabolites (Fig. 1g). Next, the kidney tissues from *db/db* mice were examined by matrix-assisted laser desorption/ionization time-of-flight mass spectrometry (MALDI-TOF MS) analysis. Spatial expression of the regional markers p-cresol sulfate, glycerylphosphorylethanolamine, cytidine, dehydroepiandrosterone, inosinic acid, adenosine 3′,5′-diphosphate, nicotinamide adenine dinucleotide (NADH), and hydrated NADH (NADHX) was visualized by MALDI-MSI (Fig. 1h). Pearson correlation analyses of metabolite biomarkers in the kidney tissues of *db/db* mice indicated a close connection (Fig. 1i). We also obtained variable importance in projection (VIP) scores in projections from partial least squares discrimination analysis (PLS-DA) to identify differentially accumulated metabolites with significant top contributions (Fig. 1j). Based on the changes of metabolites, we detected these metabolite differences in the group separation status (Fig. 1k).

### Spatially segregated metabolic multiregion dissection of diabetic mouse kidney

**Figure 2:**
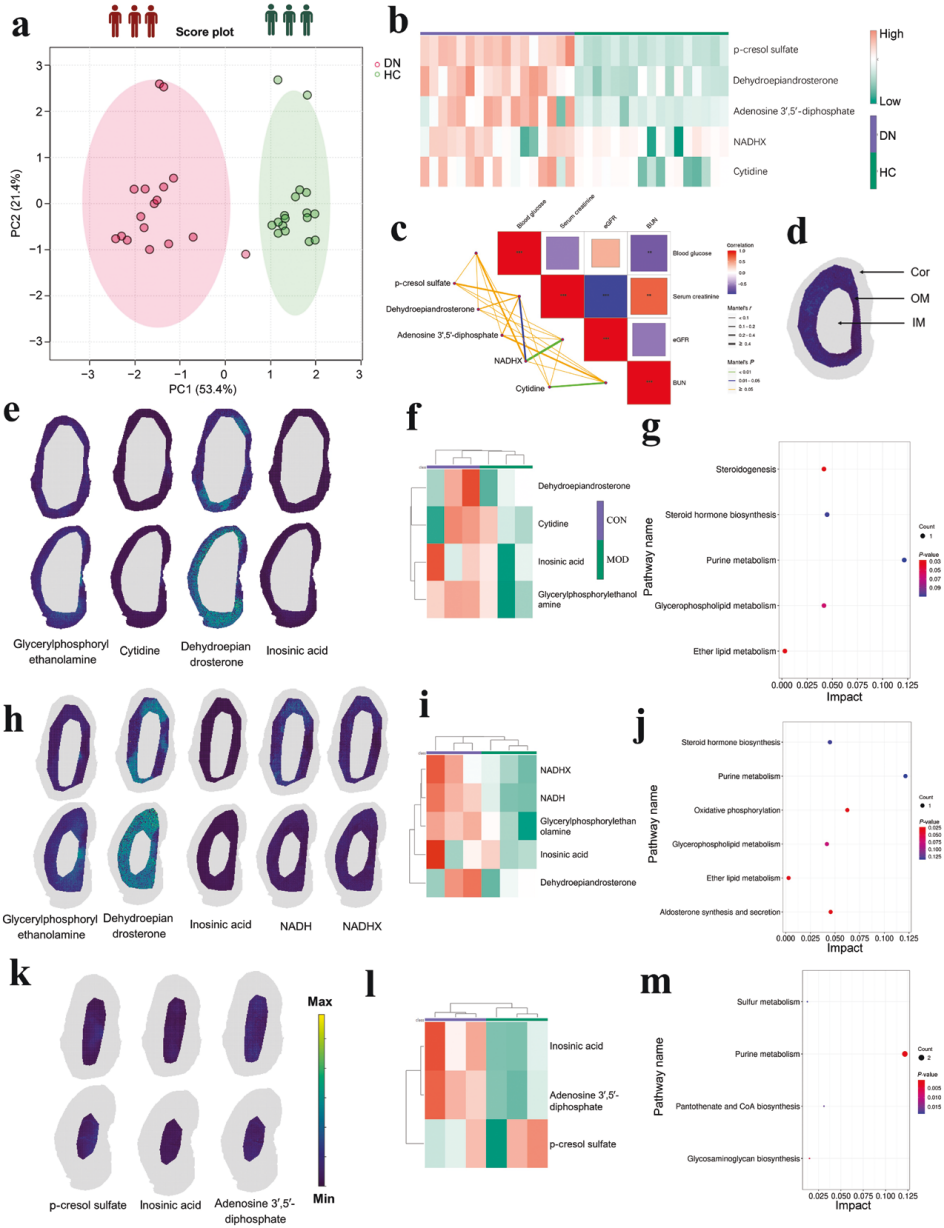
Spatially segregated metabolic multiregion dissection of diabetic mouse kidney. (a) PCA score plots of biomarker signatures in serum samples obtained from DN patients and HCs using UPLC-MS. (b) Heatmap of metabolite biomarkers showing abundance differences between DN patients and HCs. (c) Association of the metabolite levels with patient clinical features (blood glucose, serum creatinine, eGFR, and BUN). This plot displays Mantel correlations between the contents of metabolite biomarkers and clinical parameters. The color and size of the block indicate the strength of the correlation. Positive correlations are represented by red shades, while negative correlations are represented by blue shades. Blocks are used to represent statistically significant correlations (*P* < 0.05). (d) Schematic diagram of kidney anatomy structure (Cor, the kidney cortex including its vasculature; OM, the outer stripe of kidney medulla including its vasculature; IM, the inner stripe of kidney medulla including some vasculature, the main arteries, and veins within the renal parenchyma, the kidney papilla, the kidney pelvis, part of the external renal vessels, part of the ureter, and uniform surrounding tissue). (e) Localization of glycerylphosphorylethanolamine, cytidine, dehydroepiandrosterone, and inosinic acid in mouse kidney tissue Cor segmentation with higher spatial resolution applying MALDI-MSI analysis. (f) Heatmap showing the metabolites in mouse kidney tissue Cor region. (g) Pathway enrichment analysis of metabolite biomarkers associated with kidney tissue Cor region in transgenic *db/db* mice. (h) Localization and distribution of glycerylphosphorylethanolamine, dehydroepiandrosterone, inosinic acid, NADH, and NADHX in mouse kidney tissue OM segmentation with MALDI-MSI analysis. (i) Heatmap showing the metabolite levels in mouse kidney tissue OM region. (j) Pathway enrichment analysis of metabolite biomarkers associated with kidney tissue OM region. (k) Localization and distribution of p-cresol sulfate, inosinic acid, and adenosine 3′,5′-diphosphate in mouse kidney tissue IM segmentation using MALDI-MSI analysis. (l) Heatmap showing the metabolite levels in mouse kidney tissue IM region. (m) Pathway enrichment analysis of metabolite biomarkers associated with kidney tissue IM region in transgenic *db/db* mice.

Metabolic profile characteristics from DN patients and healthy controls (HCs) were analyzed using ultra-performance liquid chromatography with tandem mass spectrometry (UPLC-MS) via PCA model (Fig. 2a), and the score plot shows a significant separation trend. To visualize expression levels, the heatmap of metabolite biomarkers showed the abundance differences between DN patients and HCs (Fig. 2b). To investigate the link between metabolite levels and patient features, the correlation analysis (Mantel test) matrix was analyzed (Supplementary Table S2), and these metabolites showed a broad range of correlations with clinical biochemical parameters (blood glucose, serum creatinine, estimated glomerular filtration rate [eGFR], and blood urea nitrogen [BUN]). The levels of NADHX were positively correlated with eGFR, blood glucose, and serum creatinine; cytidine was positively correlated with BUN; p-cresol sulfate, dehydroepiandrosterone, and adenosine 3′,5′-diphosphate were positively correlated with serum creatinine (Fig. 2c). Receiver operator characteristic (ROC) diagnostic analysis demonstrated that p-cresol sulfate, dehydroepiandrosterone, and adenosine 3′,5′-diphosphate exhibited strong diagnostic potential, as evidenced by their high area under the curve (AUC) values (Supplementary Fig. S2), highlighting their utility as promising biomarkers for early detection and progression monitoring of DN. Figure 2d represents a schematic diagram of kidney anatomy structure for the kidney Cor including its vasculature, outer stripe of kidney medulla (OM) including its vasculature, the inner stripe of kidney medulla (IM) including some vasculature, the main arteries and veins within the renal parenchyma, the kidney papilla, pelvis, part of the external renal vessels, part of the ureter, and uniform surrounding tissue. We next investigated distinct region-specific metabolite patterns from localization and distribution analysis of mouse kidney tissue and found that glycerylphosphorylethanolamine, cytidine, dehydroepiandrosterone, and inosinic acid were distributed in the cortical region of mouse kidney (Fig. 2e); glycerylphosphorylethanolamine, dehydroepiandrosterone, and inosinic acid were significantly downregulated in the Cor anatomical subregion (Fig. 2f), and were mainly enriched in steroidogenesis, steroid hormone biosynthesis, purine metabolism, glycerophospholipid metabolism, and ether lipid metabolism (Fig. 2g). Glycerylphosphorylethanolamine, dehydroepiandrosterone, inosinic acid, NADH, and NADHX mainly accumulated in the OM segmentation region (Fig. 2h). These metabolites were significantly downregulated in the OM anatomical subregion of kidney tissue in *db/db* mice (Fig. 2i). They were predominantly involved in steroid hormone biosynthesis, purine metabolism, oxidative phosphorylation, glycerophospholipid metabolism, ether lipid metabolism, and aldosterone synthesis and secretion (Fig. 2j). The p-cresol sulfate, inosinic acid, and adenosine 3′,5′-diphosphate were mainly located in the IM anatomical subregion (Fig. 2k). In the IM segmentation region of kidney tissue in *db/db* mice, the levels of inosinic acid and adenosine 3′,5′-diphosphate were decreased, whereas the level of p-cresol sulfate was increased (Fig. 2l). These metabolites were mainly enriched in sulfur metabolism, pantothenate and coenzyme A (CoA) biosynthesis, purine metabolism, and glycosaminoglycan biosynthesis (Fig. 2m).

### Function-specific multiregion metabolic atlas of diabetic mouse kidney

**Figure 3:**
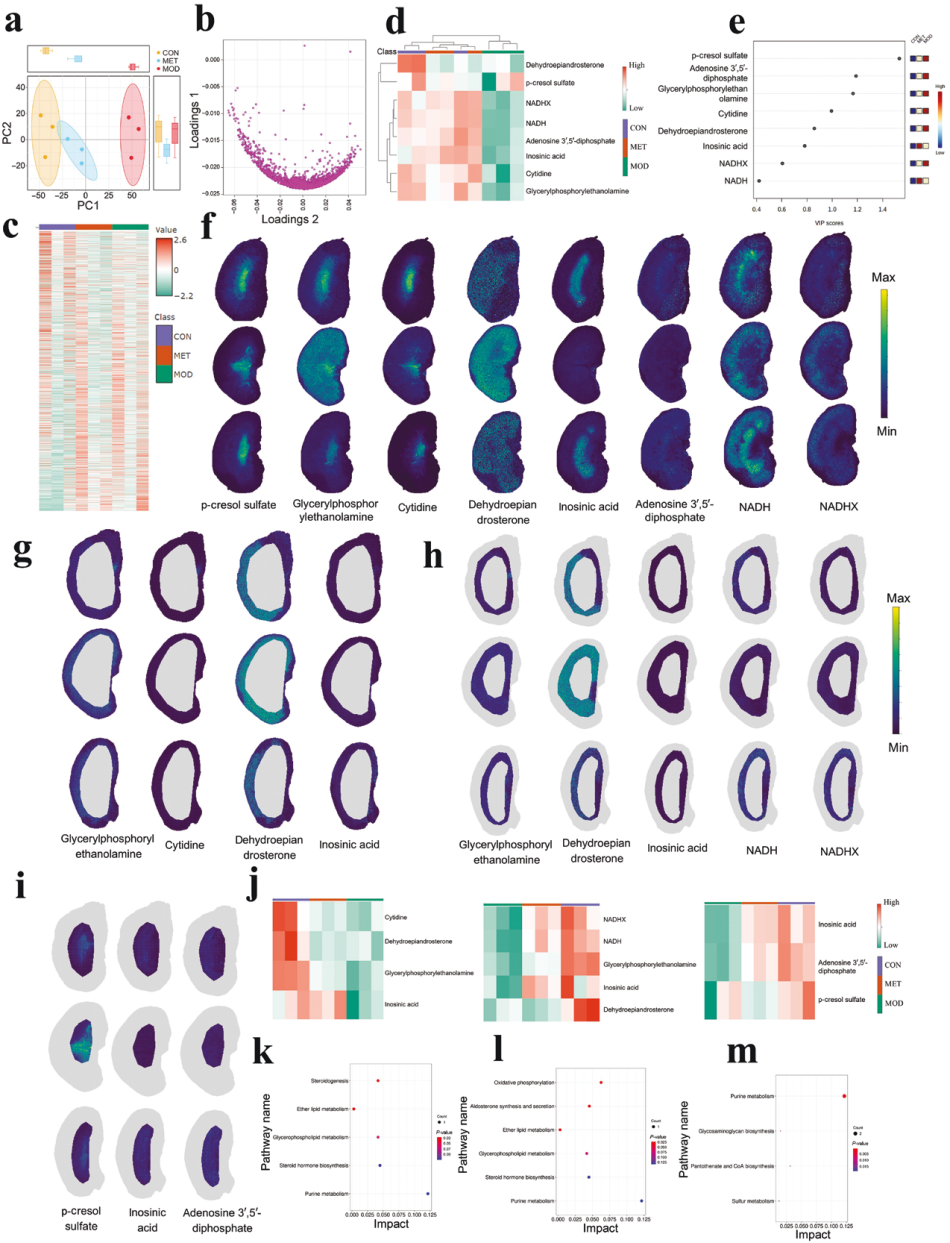
MET-induced function-specific metabolite atlas of diabetic mouse kidney by spatially resolved anatomical multiregion metabolomics. (a) PCA score plots based on the metabolite profiling of mouse kidney tissue. (b) PLS loading plot of metabolites in transgenic *db/db* mice after MET treatment. (c) Hierarchical clustering analysis of the detected ions in the mouse kidney tissue. (d) Heatmap of mouse kidney biomarkers showing abundance differences among different groups. (e) VIP scores with the metabolite biomarkers in diabetic mouse kidney samples after MET treatment. (f) MALDI-MSI analysis of mouse kidney biomarkers and their corresponding spatial distribution in kidney tissue samples from *db/dm* (up row, control group), *db/db* mice (middle row, model group), and the MET group (down row). (g) Spatial distribution of representative metabolites, including glycerylphosphorylethanolamine, cytidine, dehydroepiandrosterone, and inosinic acid, in the kidney tissue Cor segmentation from the control group, model group, and MET group. (h) MALDI-MSI analysis for the spatial distribution of metabolites, including glycerylphosphorylethanolamine, dehydroepiandrosterone, inosinic acid, NADH, and NADHX, in mouse kidney tissue OM segmentation. (i) Spatial localization and distribution of metabolites, including p-cresol sulfate, inosinic acid, and adenosine 3′,5′-diphosphate, in mouse kidney tissue IM segmentation using MALDI-MSI analysis from *db/dm* mice (up row, control group), *db/db* mice (middle row, model group), and MET group (down row). (j) Heatmap showing the metabolite levels in the Cor (left), OM (middle), and IM (right) regions in mouse kidney tissue. (k) Pathway enrichment analysis of MET-altered metabolites from the Cor region in mouse kidney tissue. (l) Pathway enrichment analysis of MET-altered metabolites from the OM region in mouse kidney tissue. (m) Pathway enrichment analysis of MET-altered metabolites from the IM region in mouse kidney tissue.

PCA score plot based on the metabolite profile of diabetic mouse kidney showed that the state of the MET group was far away from the model group and close to the control group (Fig. 3a). PLS loading plot showed the performance of distribution state of metabolites in *db/db* mice after MET treatment (Fig. 3b). Hierarchical clustering reflected MET-induced distinct metabolite profiles and clearly distinguished the overall functional regulatory role by MET (Fig. 3c). Heatmap of mouse kidney biomarkers also showed abundance differences among different groups (Fig. 3d). VIP score with the metabolite biomarkers indicated the order of importance in diabetic mouse kidney after MET treatment (Fig. 3e). The utilization of MALDI-MSI analysis could facilitate the elucidation of spatial distribution pattern of endogenous metabolite biomarkers regulated by MET (Fig. 3f). We next explored MET-induced anatomical multiregion metabolite changes of diabetic mouse kidney. It revealed that MET regulated the spatial distributions of metabolites glycerylphosphorylethanolamine, cytidine, dehydroepiandrosterone, inosinic acid in the kidney tissue Cor segmentation (Fig. 3g). MALDI-MSI analysis presented the spatial distributions of glycerylphosphorylethanolamine, dehydroepiandrosterone, inosinic acid, NADH, and NADHX in mouse kidney tissue OM segmentation from *db/dm* mice, *db/db* mice, and MET group (Fig. 3h). Spatial localization and distribution region of metabolites revealed that MET affected the levels of p-cresol sulfate, inosinic acid, and adenosine 3′,5′-diphosphate in mouse kidney tissue IM segmentation using MALDI-MSI analysis (Fig. 3i). Compared with the metabolite distribution of the model group, MET upregulated inosinic acid in the Cor anatomical subregion, and upregulated inosinic acid, NADH, and NADHX in the OM segmentation region (Fig. 3j). These metabolites were mainly enriched in pathways such as steroidogenesis, ether lipid metabolism, glycerophospholipid metabolism, steroid hormone biosynthesis, and purine metabolism (Fig. 3k). In the IM anatomical subregion, MET upregulated adenosine 3′,5′-diphosphate and inosinic acid, which were predominantly involved in oxidative phosphorylation, aldosterone synthesis and secretion, ether lipid metabolism, glycerophospholipid metabolism, steroid hormone biosynthesis, and purine metabolism (Fig. 3l). Meanwhile, in the IM segmentation region, MET downregulated p-cresol sulfate, which was mainly enriched in pathways such as purine metabolism, glycosaminoglycan biosynthesis, pantothenate and CoA biosynthesis, and sulfur metabolism (Fig. 3m), suggesting that these metabolic pathways may play a regulatory role in the MET treatment process.

### Spatial proteomics reveals MET-regulated multiregion-specific protein

**Figure 4:**
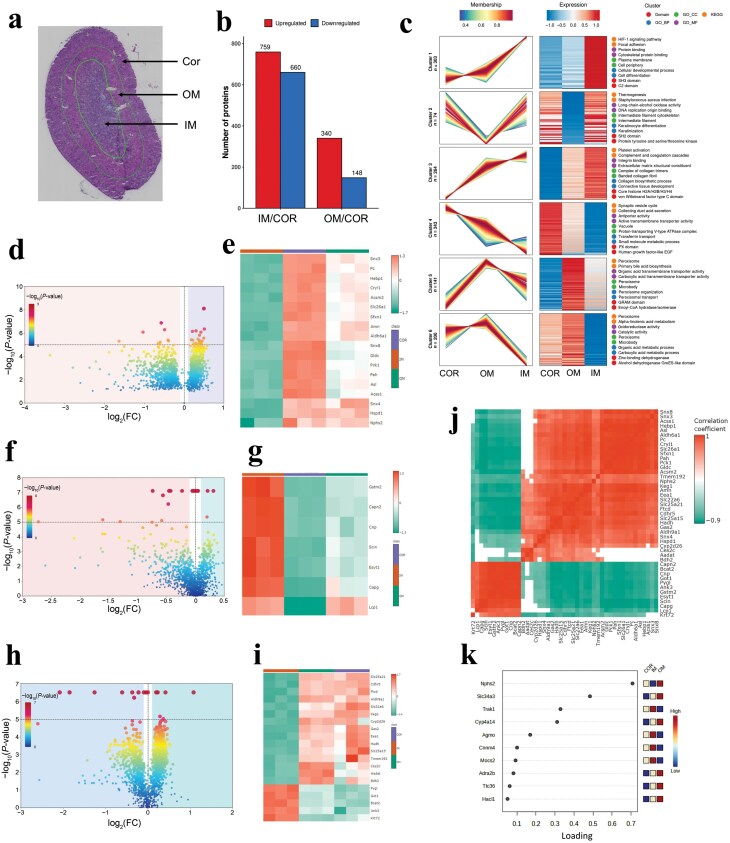
Spatial proteomics revealing MET-regulated multiregion-specific protein features of diabetic mouse kidney. (a) H&E staining showing annotation of histological kidney Cor, OM, and IM. (b) Protein changes regulated by MET in kidney tissue multiregion of transgenic *db/db* mice. (c) Summary of protein expression pattern clustering analysis based on the Fuzzy c-means algorithm analysis of GO/KEGG/Domain. (d) Heatmap illustrating the protein changes between Cor region and IM region in transgenic *db/db* mouse kidney. (e) Volcano plot showing the differentially expressed proteins between Cor region and IM region in transgenic *db/db* mouse kidney. (f) Heatmap showing the protein changes between Cor region and OM region in transgenic *db/db* mouse kidney. (g) Volcano plot comparing the alterations in differentially expressed proteins between IM region and OM region in transgenic *db/db* mouse kidney. (h) Alterations in differentially expressed proteins between Cor region and OM region in transgenic *db/db* mouse kidney. (i) Heatmap displaying the protein changes of IM region and OM region in transgenic *db/db* mouse kidney. (j) Spearman’s correlation between protein abundance and Cor, IM, and OM regions in transgenic *db/db* mouse kidney. The red color indicates a positive value and the green color indicates a negative value. (k) Top loading features of PLS-DA algorithm ranking for the top protein regulated by MET.

Hematoxylin and eosin (H&E) staining showed the annotation of histological Cor, OM, and IM (Fig. 4a). Vanquish UPLC-MS analysis resulted in the identification and quantification of 1907 proteins (Fig. 4b). Fuzzy c-means algorithm analyses of GO/KEGG/Domain were performed to gain overall proteome functional insights and reflected the distinct protein abundance expression profiles (Fig. 4c). They were predominantly involved in small molecule metabolic processes. Notably, it successfully visualized the differentially expressed proteins between the Cor region and IM region in *db/db* mouse kidney (Fig. 4d), and the heatmap illustrates the protein changes between the Cor region and IM region (Fig. 4e), such as heat shock protein family D member 1 (Hspd1), nephrosis 2 (Nphs2), and phenylalanine-4-hydroxylase (Pah), etc. Analysis of dynamic changes in the proteome revealed that the abundance of some proteins was significantly altered between the Cor region and OM region (Fig. 4f). Next, the differentially expressed proteins between the Cor and OM regions were quantified using liquid chromatography-mass spectrometry (LC/MS) to generate a heatmap of protein abundance (Fig. 4g).

The alterations of differentially expressed proteins between the IM region and OM region were compared by volcano plot (Fig. 4h) and heatmap (Fig. 4i). Interestingly, we discovered several proteins with unique localization among different regions of mouse kidney (Supplementary Tables S3−S5). Among protein alterations, we performed correlation analyses to evaluate the intrinsic relationship in the multiregion regions (Fig. 4j). Nphs2 exhibited a strong positive correlation with Cdhr5, Keg1, and Aldh6a1, and demonstrated a negative relationship with Ank3, Pygl, and Got1. VIP loading plot from the PLS-DA algorithm was used to identify differentially expressed proteins with significant contribution (Fig. 4k), and it was found that Nphs2 ranked as the top loading feature regulated by MET. Noticeably, the expression levels of NPHS2 protein in 17 individuals with DN patients were significantly increased compared with HCs (*P* < 0.01) (Supplementary Fig. S3a). The clinical performance of the NPHS2 protein was evaluated through an ROC curve using a random forest model (Supplementary Fig. S3b).

### Construction of co-expression model for kidney multiregion proteins

**Figure 5:**
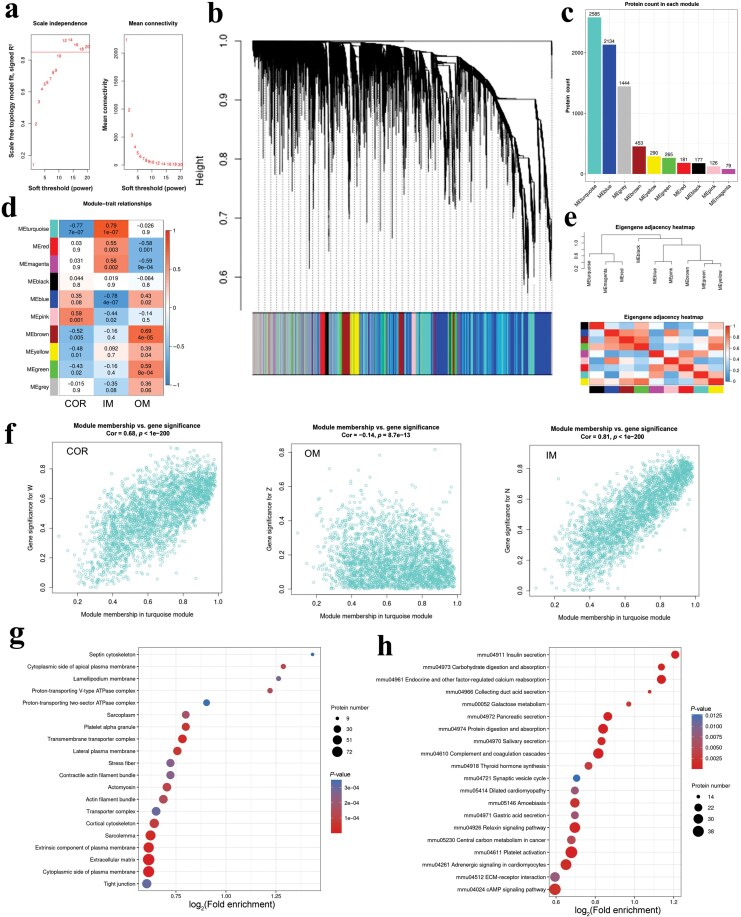
Construction of the co-expression model for the differentially expressed proteins in kidney-specific region. (a) Analysis of the soft threshold powers. (b) The clustering dendrogram based on topological overlap and specified module colors. (c) Identification of the differentially expressed proteins related to the module phenotypes. (d) Module−trait relationship diagrams generated for the co-expression model datasets. The correlation and *P*-value for each module−trait combination is displayed. (e) Clustering adjacency of module proteins. (f) Scatterplots of module membership in the turquoise module versus protein significance among each region. (g) Functional enrichment analysis in turquoise module proteins. (h) KEGG enrichment analysis of turquoise module proteins.

First, the expression levels of all proteins from diabetic mouse kidney multiregion were used to construct the co-expression modules by the weighted gene co-expression network analysis (WGCNA) algorithm for further screening of significant protein modules. When the power value achieved 10, the independence degree was 0.8, and the mean connectivity degree was higher (Fig. 5a). A total of 10 distinct protein co-expression modules were identified in diabetic mouse kidney through the dynamic WGCNA algorithm (Fig. 5b), and the column diagram also depicts the number of proteins in each module (Fig. 5c). Next, we used hierarchical clustering to identify protein modules with similar expression patterns. The proteins in these 10 modules were applied to simultaneously analyze the association among each module’s traits (Fig. 5d). Furthermore, to screen for significant protein modules, we found that the turquoise module presented the highest correlation with Cor and OM regions, containing 2585 proteins. Of note, the turquoise module had a significantly negative correlation with the Cor trait and a significantly positive correlation with the OM trait, and was selected for further analysis (*P* < 0.001). This module showed the strongest correlation with Cor and OM traits. The heatmap displays the adjacency of these feature modules (Fig. 5e). A scatter plot of protein significance versus module membership was ultimately plotted in the turquoise module (Fig. 5f). Additionally, the Gene Ontology (GO) database was used to analyze the role of turquoise module proteins. The most prevalent molecular function category was related to septin cytoskeleton and cytoplasmic side of apical plasma membrane (Fig. 5g). The insulin secretion, carbohydrate digestion and absorption, and calcium reabsorption regulated by endocrine and other factors were enriched in the turquoise module from the Kyoto Encyclopedia of Genes and Genomes (KEGG) enrichment analysis (Fig. 5h). The association of the metabolite abundance with protein levels in the Cor region (Supplementary Fig. S4a; Supplementary Table S6), OM region (Supplementary Fig. S4b; Supplementary Table S7), and IM region (Supplementary Fig. S4c; Supplementary Table S8) in response to MET treatment was analyzed via the correlation analysis, which revealed a close association between Nphs2 and glycerylphosphorylethanolamine, inosinic acid, dehydroepiandrosterone, and cytidine. Next, we delved into the pathway enrichment characteristics for each region by comparing their differential expression patterns in detail. The significantly enriched KEGG signaling pathways appear to show distinct differences between these three regions. Purine metabolism and ether lipid metabolism pathways were enriched in the Cor region (Supplementary Fig. S4d; Supplementary Table S9), and OM region (Supplementary Fig. S4e; Supplementary Table S10); at the same time, the pathways of pantothenate and CoA biosynthesis were mainly enriched in the IM region (Supplementary Fig. S4f; Supplementary Table S11) in response to MET treatment. In addition, we performed the gene set enrichment analysis (GSEA) to reveal the relevant biological functions, and found that MET-related biological processes, such as the pathways of pantothenate and CoA biosynthesis, purine metabolism, ether lipid metabolism, were activated in response to MET treatment (Supplementary Fig. S4g), indicating that these metabolic pathways may play an important role in the MET treatment.

### Integrated functional analysis reveals MET-modulated metabolism pattern

**Figure 6:**
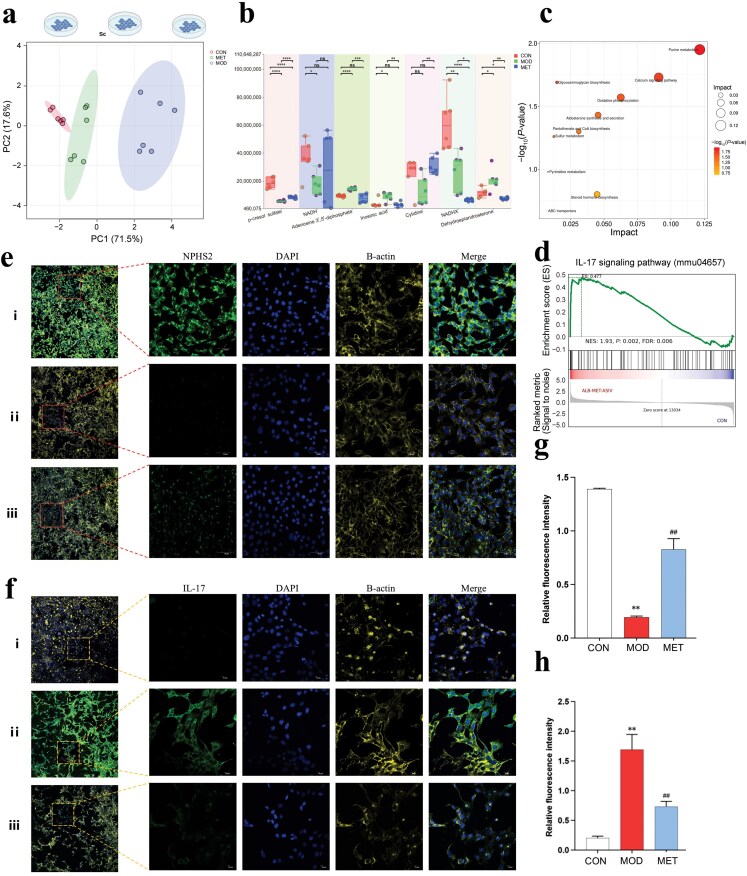
Integrated functional analysis revealing MET-modulated metabolism pattern in MPC-5 cells. (a) PCA for MET-affected intracellular metabolism of MPC-5 cells. (b) Box line diagram of differential metabolites for multiple group comparison. (c) Pathway enrichment analysis of MET-altered metabolites in MPC-5 cells. (d) GSEA analysis of pathway enrichment for cellular transcriptome. (e) Immunofluorescence detection of MET-mediated NPHS2 in MPC-5 cells (10 × 40x, scale bar = 50 μm). (f) Immunofluorescence detection of MET-impacted IL-17 (10 × 40x, scale bar = 10 μm). i, CON group; ii, MOD group; iii, MET group. (g) Relative fluorescence intensity of NPHS2 in MPC-5 cells. (h) Relative fluorescence intensity of IL-17 in MPC-5 cells. Note: i, *db/dm* mice; ii, *db/db* mice; iii, MET group. ^**^ represents comparison with the control group. *P* < 0.01; ^##^ represents comparison with the model group, *P *< 0.01.

As shown in Supplementary Fig. S5a, we obtained the representative mouse kidneys of the *db/dm*, *db/db*, and MET groups. Compared to the control group, the model group presented significantly elevated levels of fasting blood glucose (FBG) (Supplementary Fig. S5b), homeostasis model assessment of insulin resistance (HOMA-IR) (Supplementary Fig. S5c), and kidney weight (Supplementary Fig. S5d) in *db/db* mice (*P* < 0.001). After treatment, the MET group presented a significant decrease in the levels of FBG (*P* < 0.001), HOMA-IR (*P* < 0.05), and kidney weight (*P* < 0.05) compared with the mode group. At the end of the experiment, 4 weeks after MET treatment, no apparent histologic changes indicative of *db/db* mouse kidneys were observed based on Masson staining, periodic acid-Schiff (PAS) staining, periodic acid-silver methenamine (PASM) staining, picrosirius red (PSR) staining, and Oil Red O staining images (Supplementary Fig. S5e). Furthermore, our findings suggested that MET had a beneficial effect on preventing kidney injury in *db/db* mice. The MET-modulated metabolism pattern in MPC-5 cells was explored by an integrated functional analysis. PCA showed segregation of the control and MET groups from the model group (Fig. 6a). MET samples were distributed over the model group, clustering closest to the control group. As shown in Fig. 6b, compared to the model group, the MET group exhibited significantly elevated levels of p-cresol sulfate, inosinic acid, and cytidine in MPC-5 cells. Adenosine 3′,5′-diphosphate and dehydroepiandrosterone levels were significantly increased in the model group, and after treatment with MET, it exhibited a significantly lower level than the model group. These regulated metabolites were enriched in purine metabolism, pantothenate and CoA biosynthesis, sulfur metabolism, pyrimidine metabolism, steroid hormone biosynthesis, and so on (Fig. 6c). Transcriptome sequencing analysis further uncovered the relationships of MET-mediated cellular function, and GSEA analysis indicated that MET effect was highly positively correlated with interleukin-17 (IL-17) signaling pathway (Fig. 6d). Immunofluorescence assay was used to verify the effects of MET on the expression of NPHS2 (Fig. 6e) and IL-17 (Fig. 6f) in MPC-5 cells. MET significantly increased the expression of NPHS2 in MPC-5 cells (*P* < 0.01) (Fig. 6g), while MET intervention significantly reduced the expression of IL-17 (*P* < 0.01) (Fig. 6h).

## Discussion

Metabolism plays a crucial role in maintaining tissue homeostasis, function, and therapeutic ability. Spatial metabolomics provides great hope to advance our fundamental understanding of tissue biology and identify potential treatment measures within tissue complexity [22]. Recently, scientists have become interested in the pharmacological effects of MET on the kidneys [23]. In particular, the effects of MET against DN have been explored, and it has been shown that MET can inhibit renal inflammation, oxidative stress, and fibrosis, regulate metabolic function, and normalize renal function parameters. However, the metabolic characteristics of MET-mediated function-specific metabolism in the kidney microregion remain obscure. Although some studies provide substantial insights into the disease process, their mapping of kidney tissue is often limited to a single metabolic and protein expression pattern.

Here, we present a comprehensive study of the metabolomics and proteomics in combination with MALDI-MSI to unravel functional characterization alterations of the anatomic multiregion of diabetic mouse kidney. In addition, spatially resolved multiregion and function-specific metabolite and protein atlas of the diabetic mouse kidney were employed to compare and reveal the relevant alterations in metabolite distribution. The expression pattern *t*-SNE, UMAP, and PCA analyses of spatial metabolic characterization of diabetic mouse kidney differed in *db/dm* and *db/db* mice. Spatial metabolomics by MALDI-MSI analysis identified p-cresol sulfate, glycerylphosphorylethanolamine, cytidine, dehydroepiandrosterone, inosinic acid, adenosine 3′,5′-diphosphate, NADH, and NADHX as metabolite biomarkers that correlate with mouse kidney segments selected from Z-score analysis. These metabolites had a close correlation with clinical biochemical parameters from DN patients. We next explored the distinct region-specific patterns from metabolite localization and distribution analysis of mouse kidney tissue. Spatially metabolic multiregion analysis revealed that glycerylphosphorylethanolamine, cytidine, dehydroepiandrosterone, and inosinic acid were distributed in Cor anatomical subregion, and mainly enriched in steroidogenesis, steroid hormone biosynthesis, purine metabolism, glycerophospholipid metabolism, and ether lipid metabolism. Glycerylphosphorylethanolamine, dehydroepiandrosterone, inosinic acid, NADH, and NADHX also accumulated in the OM anatomical subregion, and predominantly involved in steroid hormone biosynthesis, purine metabolism, oxidative phosphorylation, glycerophospholipid metabolism, ether lipid metabolism, and aldosterone synthesis and secretion. The p-cresol sulfate, inosinic acid, and adenosine 3′,5′-diphosphate were located at the IM anatomical subregion of kidney tissue and mainly enriched in sulfur metabolism, pantothenate and CoA biosynthesis, purine metabolism, and glycosaminoglycan biosynthesis. These findings highlight unique metabolic characteristics associated with DN, which may provide insights into potential metabolic patterns and therapeutic targets.

The multiple renal protective effects of MET have been widely studied, including inhibiting renal inflammation, oxidative stress, and fibrosis [24, 25]. MET modulates dysregulated metabolic pathways by stimulating the metabolic defense system, normalizes renal function parameters, reduces histopathological changes, and is beneficial for protecting the kidneys. The MET group presented a significant decrease in the levels of FBG, HOMA-IR, and kidney weight compared with *db/db* mice, and no apparent histologic changes indicative of *db/db* mouse kidney were observed, which suggested that MET could ameliorate the pathological and biochemical indicators of *db/db* mice. The anatomical multiregion metabolic changes of diabetic mouse kidney were explored by the spatially resolved metabolomics. Notably, we found that MET impacted the spatial metabolite distributions of glycerylphosphorylethanolamine, cytidine, dehydroepiandrosterone, and inosinic acid in the Cor segmentation, regulated the spatial distributions of glycerylphosphorylethanolamine, dehydroepiandrosterone, inosinic acid, NADH, and NADHX in the OM segmentation, and affected p-cresol sulfate, inosinic acid, and adenosine 3′,5′-diphosphate in the IM segmentation. Specifically, compared with the distribution and expression of metabolites in the model group, MET upregulated inosinic acid, NADH, and NADHX in the Cor and OM regions, which were mainly enriched in steroidogenesis, ether lipid metabolism, glycerophospholipid metabolism. In the IM region, MET upregulated adenosine 3′,5′-diphosphate and inosinic acid, and conversely, downregulated p-cresol sulfate, which is linked to purine metabolism, pantothenate, and CoA biosynthesis, etc. These changes exhibited the region-specific metabolite patterns. Additionally, alterations in these metabolites related to distinct regions suggest the potential and robustness of our platform in identifying region-specific distribution characteristics.

To add a spatial dimension to explore the anatomical multiregion metabolic changes of diabetic mouse kidney, we utilized spatial proteomics for the analysis of protein localization based on region abundance profiles to reveal MET-regulated specific protein features according to the annotation of histological Cor, OM and IM. Next, a total of 1907 proteins were quantified, which were involved in small molecule metabolic processes. To investigate the spatial identification of differently expressed proteins among different regions of the three sections (Cor, OM, and IM), we calculated their expression by unsupervised clustering of the spatial distribution of proteins. We compared in detail the number of proteins expressed differently in each kidney region and discovered several proteins with unique localization among different regions in mouse kidney. Interestingly, correlation analysis was performed to evaluate the intrinsic relationship in kidney multiregion and demonstrated that Nphs2 could exhibit a strong association among different regions. Noticeably, PLS-DA algorithm identified that Nphs2 was the highest loading feature regulated by MET.

Kidney function is highly dependent on spatial organization and corresponding marker proteins, therefore detecting region-specific proteins is crucial to help understand the complexity and pathogenesis of the kidney. We further examined functional enrichment analysis of the proteins from multiregion of diabetic mouse kidney and conducted the co-expression model by the WGCNA algorithm for the differentially expressed proteins. As expected, 10 distinct protein co-expression modules in diabetic mouse kidney were identified via the WGCNA algorithm. From the KEGG enrichment analysis, insulin secretion, carbohydrate digestion and absorption, and calcium reabsorption regulated by endocrine and other factors were enriched in the turquoise module. Next, pathway enrichment characteristics for each region were revealed in detail. Purine metabolism and ether lipid metabolism pathways were enriched in the Cor and OM regions, while pantothenate and CoA biosynthesis pathway were mainly enriched in the IM region by MET treatment. The results suggested that these molecular pathway changes in different segments may be an important marker of diabetes-induced renal dysfunction. In addition, the correlation analysis was performed on the metabolites and protein alterations in different kidney-specific regions, revealing a close association between Nphs2 and glycerylphosphorylethanolamine, inosinic acid, dehydroepiandrosterone, and cytidine. Integrated functional analysis revealed that MET modulated metabolism pattern in MPC-5 cells, which were enriched in pathways such as purine metabolism, pantothenate and CoA biosynthesis, sulfur metabolism, pyrimidine metabolism, steroid hormone biosynthesis, and so on. Immunofluorescence assay showed that MET could significantly increase the expression of NPHS2 in MPC-5 cells.

MET exerts region-specific effects of spatial metabolites in diabetic mouse kidneys by restoring dysregulated metabolic pathways and coordinating protein−metabolite interactions. In the Cor part, MET upregulates inosinic acid, glycerylphosphorylethanolamine, and dehydroepiandrosterone, enhancing purine metabolism, glycerophospholipid/ether lipid metabolism, and steroid hormone biosynthesis. Within the OM, MET elevates inosinic acid, NADH, and NADHX, reactivating oxidative phosphorylation, aldosterone synthesis, and carbohydrate metabolism. In the IM, MET suppresses p-cresol sulfate, while increasing adenosine 3′,5′-diphosphate and inosinic acid, thereby normalizing sulfur metabolism, pantothenate/CoA biosynthesis, and glycosaminoglycan biosynthesis. These spatial adjustments correlate with improved renal function, including reduced serum creatinine and blood glucose levels. Proteomic analysis reveals MET’s coordination with key proteins, notably upregulating NPHS2, which stabilizes glomerular integrity and correlates with metabolite restoration in the Cor. WGCNA algorithm identified a turquoise module enriched in insulin secretion and calcium reabsorption, linking MET-regulated proteins (Hspd1 and Aldh6a1) to metabolic recovery in the Cor/OM regions. MET alleviates hyperglycemia, insulin resistance, and kidney hypertrophy in *db/db* mice and suppresses pro-inflammatory IL-17 signaling in MPC-5 cells, mitigating renal injury. By spatially reprogramming metabolites, restoring energy homeostasis, and synergizing with protein networks, MET mitigates DN through multiregion metabolic repair and anti-inflammatory effects.

In this work, we first optimized a workflow for the interrogation of spatial metabolomics and proteomics, and localized the metabolic signature of anatomic multiregion to spatial protein spots of diabetic mouse kidney, providing a detailed picture and molecular composition of mouse kidney. Combining spatial metabolomics with proteomic analysis can comprehensively study various metabolic pathways in complex tissues. Most importantly, we applied this platform to understand the potential pathophysiological mechanisms of metabolic disorders in mouse kidney and created a high-resolution map describing the metabolic trajectory of MET intervention in the development of DN disease. From such an integrated platform for multiscale molecular imaging, we can explore key functions that could help define the states of spatial anatomic regions when they are perturbed. Mapping these changes will provide a new framework for region-specific features of kidney diseases. Moreover, extending anatomical structure analysis in experimental models can gain a detailed understanding of the underlying mechanisms and the identification of potential biomarkers for DN, which can provide more comprehensive features for tissue research of patients in the future. Remarkably, the comprehensive analysis process we have established can reveal the hidden molecular functional patterns generated by the relationships between these multi-molecular spatial levels with different characteristics.

Spatial metabolomics offers a transformative approach to deciphering tissue complexity by enabling precise, region-specific mapping of metabolites and their associated pathways within anatomical subregions. By integrating MALDI-MSI with multiomics analyses, it reveals spatially resolved metabolic biomarkers linked to DN and correlates them with clinical biochemical parameters, providing critical diagnostic and therapeutic insights. This technique uncovers unique metabolic patterns across kidney microregions, such as purine metabolism in Cor/OM and pantothenate/CoA biosynthesis in IM, while elucidating therapeutic mechanisms of MET intervention, which modulates region-specific metabolites to restore dysregulated pathways. Combined with proteomics, spatial metabolomics identifies key protein−metabolite interactions and co-expression networks, bridging molecular changes to functional outcomes. Its high-resolution profiling resolves tissue heterogeneity, maps metabolic trajectories, and highlights region-specific therapeutic targets, offering a robust platform to unravel pathophysiology, validate drug effects, and advance precision medicine in complex diseases.

## Conclusion

In this study, we present a comprehensive study of spatial multiregion and function-specific metabolite and protein atlas to localize the metabolic signatures and zonate microstructural proteins, which aimed to unravel functional characterization alterations of anatomic multiregion of diabetic mouse kidney and highlight the therapeutic relevance of metabolism regulated by MET. Based on anatomic location, spatial metabolomics identified eight region-specific metabolite biomarkers from mouse kidney segments, which were closely correlated with clinical biochemical parameters. Specifically, we found that MET affected spatial distribution characteristics of these regional biomarkers with segmentation localization and then successfully validated through immunofluorescence assay. The differentially expressed proteins  in each region were compared in detail, and it was found that MET-regulated Nphs2 was the highest loading feature. WGCNA algorithm identified 10 distinct protein co-expression modules in the Cor and medulla of diabetic mouse kidney. There were significant region-specific changes in the enrichment characteristics of metabolic pathways in response to MET treatment. In summary, we have developed an integrated workflow and a comprehensive multi-scale map of the kidney region profile, which directly identifies function-specific metabolites and proteins in diabetic kidney tissues and reveals their spatial distribution, providing new avenues for exploring the anatomical basis of the kidney.

## Limitations of the study

Although this study generated abundant data in molecular analysis and imaging of mouse kidneys, our study has limitations due to the limited sample size in the mouse model, and we only focused on a selected small number of mouse kidneys. Furthermore, for ethical reasons, we are unable to obtain kidney samples from healthy individuals for comparison with pathological conditions. The future improvement of this method will open up a path for incorporating spatial omics into clinical environments to help promote the application of personalized treatment or disease biomarker discovery in the future. Anyway, the spatial multiomics method provides the ability to map the spatial distribution of metabolites and proteins on renal tissue slices and may contribute to our understanding of pathological biology. This important advantage enables different analytes and anatomical information to be co-located, potentially providing researchers with a comprehensive understanding of the spatial distribution of metabolic molecules while maintaining the integrity of tissue spatial structure. In addition, integrating this spatial multiomics data enhances the molecular characteristics of tissues and shows great potential for future disease research.

## Materials and methods

### Animal experiments

C57BL/KsJ-*db/dm* and C57BL/KsJ-*db/db* male mice aged 7 weeks were obtained from Jiangsu Jicui Yaokang Biotechnology Co., Ltd., China (SCXK-2023-0009). This study was conducted for 4 weeks and the mice were divided into three groups: *db/dm* (control, normal saline-treated), *db/db* (model, normal saline-treated), and MET group, respectively. MET was administered based on the human-recommended daily dose of 65 mg/kg for the consecutive 4 weeks, while control and model group mice received no drug at all. Two hours after administration, blood was collected from the mice by enucleation for biochemical indicator detection. Mouse kidney was immediately extracted, rapidly frozen with liquid nitrogen, and subsequently stored in a −80°C refrigerator for a long time, until sectioning. This study was conducted according to the Institutional Animal Care and Use Committee of Hainan Medical University (Number: HYLL-2023-457).

### Biochemical analysis and tissue histology

FBG was determined by the blood glucose meter (Basel, Switzerland). Glycated hemoglobin (HbA1c) was detected using a glycated hemoglobin assay kit. The insulin levels were determined using Mouse Insulin ELISA Kit. To analyze the histopathological damage, the Masson staining, PAS staining, PASM staining, PSR staining, and Oil Red O staining were used to observe pathological changes in kidney tissue through Image-Pro Plus 6.0 (Media Cybemetics, U.S.A).

### Embedding procedure of mouse kidney

For cryosectioning, kidney tissues were embedded in 2% carboxymethyl cellulose (CMC) medium (10 g CMC/490 mL water, mixed overnight). Samples were placed in embedding molds, covered with CMC, and flash-frozen in isopentane cooled with dry ice (−78°C) until solidified. Embedded blocks were stored at −80°C.

### Kidney tissue sectioning

For cryosectioning, kidney tissues were equilibrated at −20°C for 30 min after transfer from −80°C storage. All tools (slides, brushes, and tweezers) were pre-chilled in the cryostat chamber (LEICA-CM1950, chamber: −20°C, sample head: −14°C). Samples were mounted using the optimal cutting temperature (OCT) compound, frozen on the Peltier stage for 5 min, and secured to the microtome. After trimming, 10-μm sections were cut and collected on indium tin oxide (ITO)-coated slides. All procedures were performed in the temperature-controlled chamber to prevent sample degradation.

### Matrix coating

Dry tissue sections mounted on ITO slides were sprayed with HTX TM spray containing 10 mg/mL 9-aminoacridine (9AA) dissolved in ethanol:water (7:3, v/v). The spray temperature was set at 90°C, the flow rate was 120 μL/min, the nitrogen pressure was 10 psi, and the spray duration was 5 min. The substrate was applied onto the glass slide four times, with a drying time of 10 s between each application.

### MALDI-MSI

MALDI-MSI analysis was performed using a Bruker TimsTOF flex system equipped with a 10 kHz 3D laser (70% constant power). Data acquisition in negative ion mode covered *m/z* 150−1200 with 30 μm spatial resolution (400 laser shots/spot). Signal intensities were normalized by root mean square method.

### MALDI-MSI data processing and analysis

Raw imaging data were processed in SCiLS™ Lab 2024 (baseline correction, peak alignment, smoothing, RMS normalization). Metabolites were annotated using METASPACE (HMDB/LipidMaps/KEGG; false discovery rate (FDR) < 10%). Multivariate analysis included *t*-SNE and UMAP for structural visualization, complemented by Pearson/cosine correlation for spatial pattern matching. Z-score analysis identified significant metabolite differences across tissue regions.

### Frozen section, laser microdissection, and trypsin hydrolysis for spatial proteomics

Kidney tissues were washed with PBS, flash-frozen in isopentane-liquid nitrogen, and embedded in the OCT compound. H&E-stained sections (5 min at 37°C) were scanned (OLYMPUS VS200, 20×) for laser microdissection (Zeiss PALM). Target regions were isolated using laser capture microdissection (LCM) and collected in microtubes at −80°C. Microdissected tissues were lysed (ultrasonication, 95°C/10 min), followed by tryptic digestion (10 ng/μL, 37°C/overnight). Proteins were reduced (5 mmol/L dithiothreitol (DTT), 56°C/30 min) and alkylated (11 mmol/L indole-3-acetic acid (IAA), RT/15 min/dark) prior to LC-MS analysis.

### LC-MS/MS analysis

Peptide samples were analyzed using a Vanquish-Neo UHPLC system with a binary solvent gradient (A: 0.1% formic acid in water; B: 0.1% formic acid in 80% acetonitrile) at a flow rate of 200 nL/min. Following chromatographic separation, peptides were ionized via NSI (1900 V) and analyzed on an Orbitrap-Astral platform (MS1: Orbitrap; MS2: Astral). Data-independent acquisition (DIA) spectra were processed using DIA-NN v1.8 against a mouse proteome database (UniProt Mus_musculus, 17,132 entries) with tryptic digestion parameters and fixed modifications. FDR was controlled at < 1% for all identifications.

### Bioinformatics analysis

Protein function annotation was conducted using multiple databases (KEGG, GO, STRING, Reactome, and WikiPathways). Differential expression analysis employed Fisher’s exact test (fold change > 1.5, *P* < 0.05) for pairwise comparisons and analysis of variance (ANOVA) for multi-group analyses. Protein clusters were identified via mFuzz clustering. Pathway enrichment was assessed through GSEA, calculating normalized enrichment scores and *P*-values to evaluate pathway associations.

### WGCNA

The WGCNA algorithm identified co-expressed protein modules using an optimal soft-threshold power to construct a weighted adjacency matrix. Hierarchical clustering grouped proteins with similar expression patterns into functional modules. Key disease-associated modules were selected for further analysis of their molecular functions.

### Serum and urine metabolite analyses from clinical participants

Serum and urine samples from DN patients and HCs were taken from the First Affiliated Hospital of Hainan Medical University. The bio-samples from clinical participants were collected and stored at −80°C. Subsequently, targeted metabolite analysis was performed using Vanquish UPLC combined with high-resolution Orbitrap Exploris mass spectrometer (Thermo Fisher Scientific, USA). It was approved by the Ethics Committee of the First Affiliated Hospital of Hainan Medical University (2023-KYL-244). Patient characteristics can be found in the supporting information (Supplementary Table S12).

### Intracellular metabolite analysis of MPC-5 cells

MPC-5 cells (Shanghai Jinyuan Biotech) were cultured in DMEM/10% FBS (37°C, 5% CO_2_) and treated under three conditions: control, 60 mmol/L high glucose (model), and high glucose plus 100 mmol/L MET. After 24 h, the cells were washed with cold PBS, flash-frozen in liquid nitrogen, and stored at −80°C. Metabolites were extracted and analyzed by LC-MS (Vanquish system) using a 12-min gradient (0.1% formic acid in water/acetonitrile) at 0.3 mL/min. MS parameters included dual-polarity detection, 325°C capillary temperature, and *m/z* 100−1000 scan range.

### Molecular docking and simulation analysis

Gromacs2022 program was used to run molecular dynamics simulations, while the TIP3P water model was used for protein simulations. The molecular dynamics simulation platform was used to dock compound−protein complexes through root mean square fluctuations, root mean square deviations, principal component trajectories, buried surface area, free energy landscape, and surface embedding structure analysis. Molecular docking binding activity ≤ −5 kcal/mol showed better performance.

### Enrichment analysis

Differential molecules were submitted to the MetaboAnalyst 6.0 platform for bioinformatics function annotation and enrichment analysis. Using GO and KEGG enrichment analyses, the differentially accumulated metabolites and proteins between different groups were studied to further elucidate their biological and cytological roles and related pathways. Based on the metabolic expression profile dataset, we performed GSEA to investigate differences in biological processes.

### Correlation and statistical analysis

Unsupervised PCA, supervised PLS-DA analysis, and hierarchical heatmap clustering analysis were used for inter-group comparison. VIP scores in projections were obtained from PLS-DA to identify differentially accumulated molecules with significant contributions. To determine the trend of expression changes in different kidney regions after MET treatment, a correlation matrix was used to describe it. The Pearson correlation coefficient was calculated based on the expression matrix of each kidney region. ANOVA was used for multiple groups followed by *t*-test. All statistical *P*-values are bilateral, and *P* < 0.05 is considered statistically significant.

## Supplementary Material

loaf019_suppl_Supplementary_Figures_S1-S5_Tables_S1-S12

## Data Availability

The authors confirm that all the data supporting the findings of this study are available within the supplementary material and corresponding authors.
