# Supplementary material for: Spatially segregated multiomics decodes metformin-mediated function-specific metabolic characteristics in diabetic kidney disease
Source: Life Metab. 2025 May 30;4(5):loaf019. doi: 10.1093/lifemeta/loaf019 (PMC13122360; doi:10.1093/lifemeta/loaf019)
Supplement: loaf019_suppl_Supplementary_Figures_S1-S5_Tables_S1-S12 [file loaf019_suppl_supplementary_figures_s1-s5_tables_s1-s12.docx]

Supplementary material for

**Spatially segregated multiomics decodes metformin-mediated functional-specific metabolic characteristics in diabetic kidney disease**

Shi Qiu^1^, Dandan Xie^1^, Sifan Guo^1^, Zhibo Wang^1^, Xian Wang^1^, Ying Cai^1,2^, Chunsheng Lin^2^, Hong Yao^3^, Yu Guan^2^, Qiqi Zhao^2^, Qiang Yang^2^, Yiqiang Xie^1,*^, Songqi Tang^1,*^, Aihua Zhang^1,2,*^

^1^International Advanced Functional Omics Platform, Scientific Experiment Center, Public Research Center, Hainan Medical University, Haikou, Hainan 571199, China

^2^Graduate School, Second Affiliated Hospital, Heilongjiang University of Chinese Medicine, Harbin, Heilongjiang 150040, China

^3^First Affiliated Hospital, Harbin Medical University, Harbin, Heilongjiang 150040, China


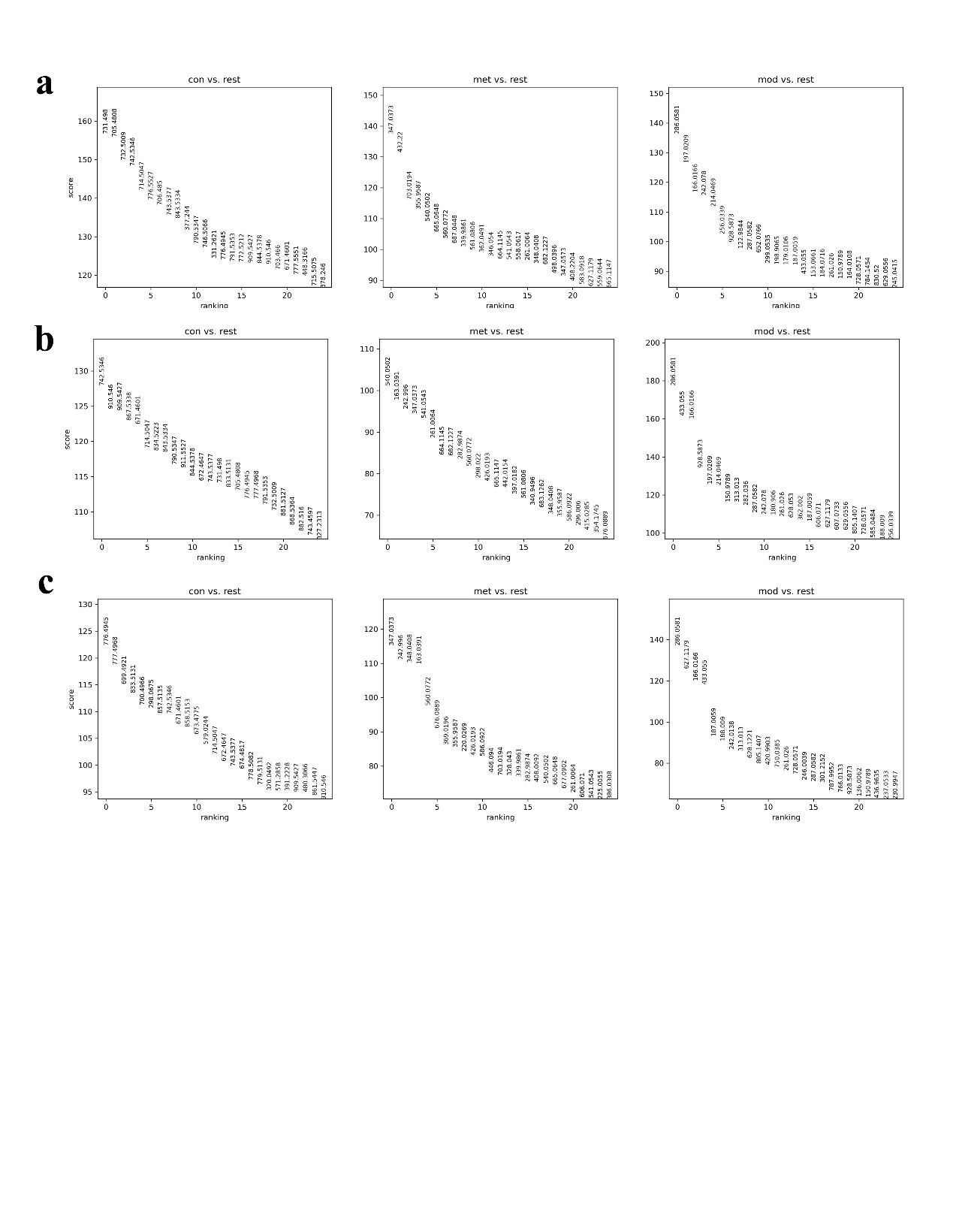


**Supplementary Figure S1** Matrix diagram on the selection analysis of metabolite biomarkers in *db/db* mouse kidney tissues. (a) Z-score analysis of ion variation associated with kidney tissue (Cor) region in transgenic *db/db* mice. (b) Z-score analysis of ion ranking in mouse kidney tissue (OM) region. (c) Z-score analysis of ion changes associated with *db/db* mouse kidney tissue (IM) region.


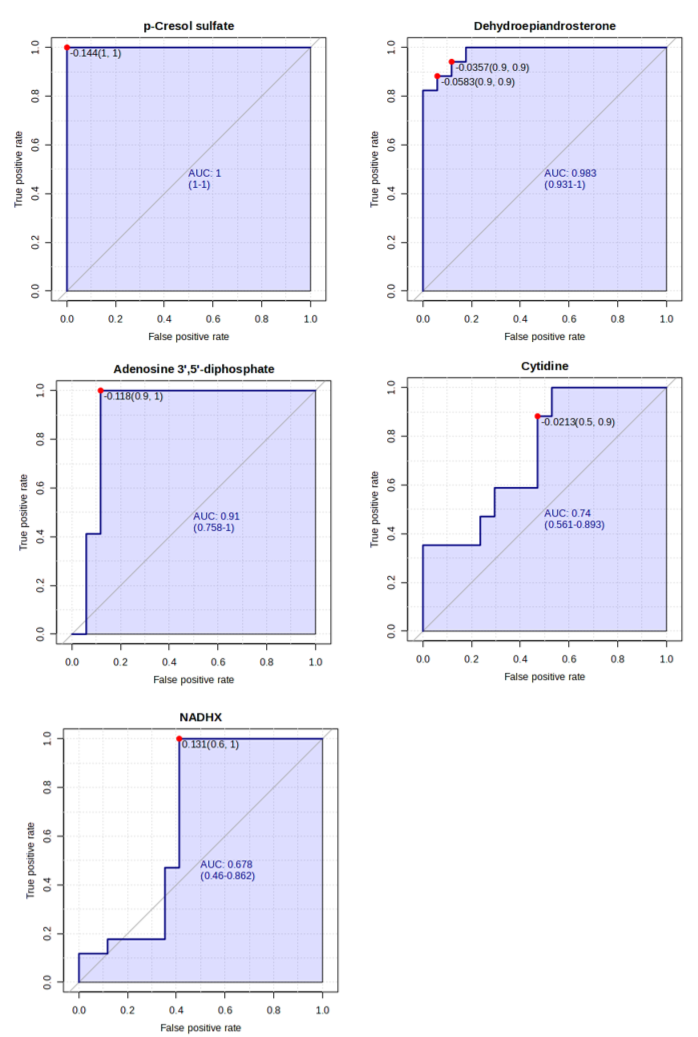


**Supplementary Figure S2** The ROC analysis of serum metabolite biomarkers in DN patients.


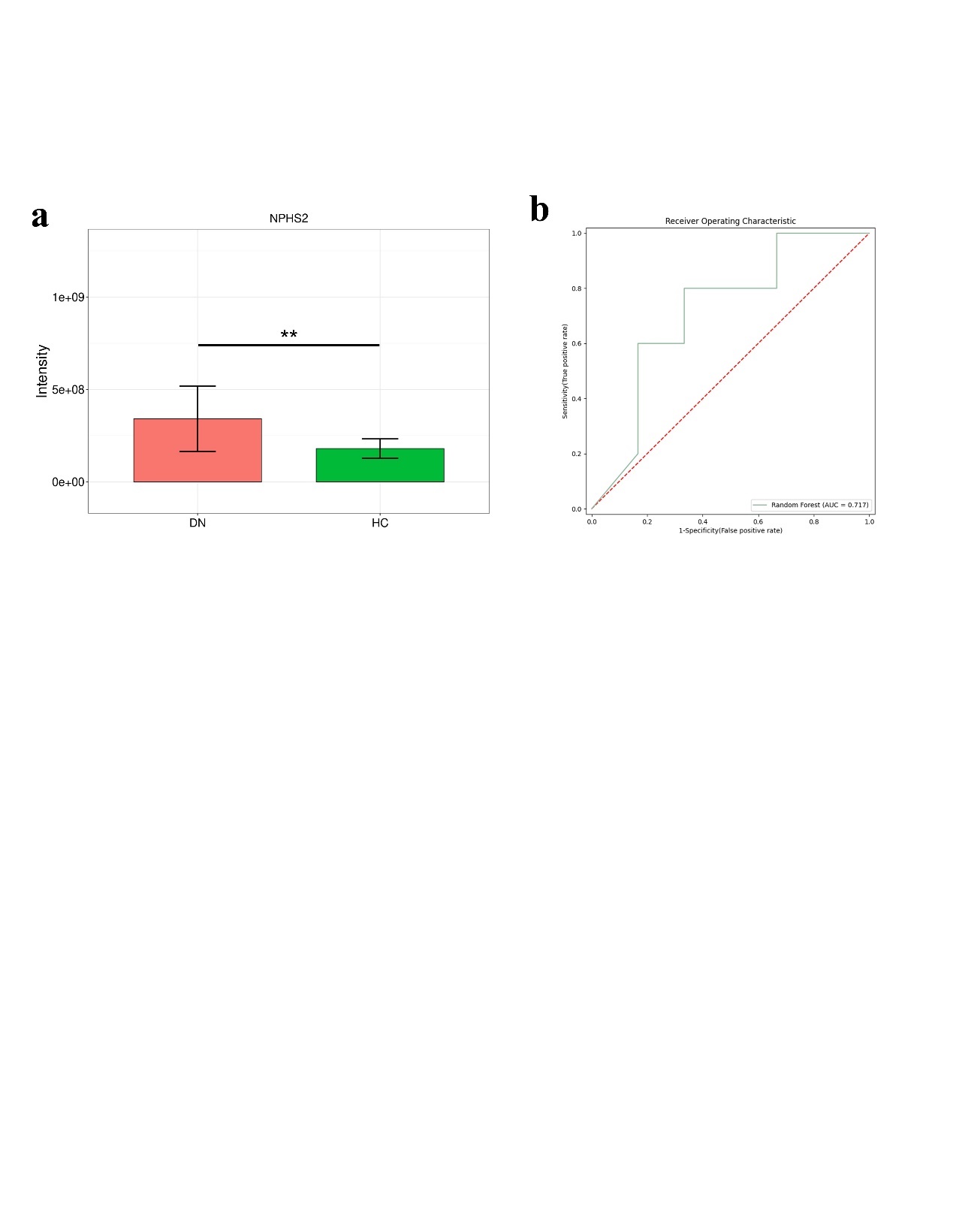


**Supplementary Figure S3** The expression level and ROC analysis of NPHS2 protein in DN patients. (a) Statistical comparison of expression level of NPHS2 protein between DN patients and HCs. (b) ROC curve analysis of random forest for discriminatory power of NPHS2 protein between DN patients and HCs.


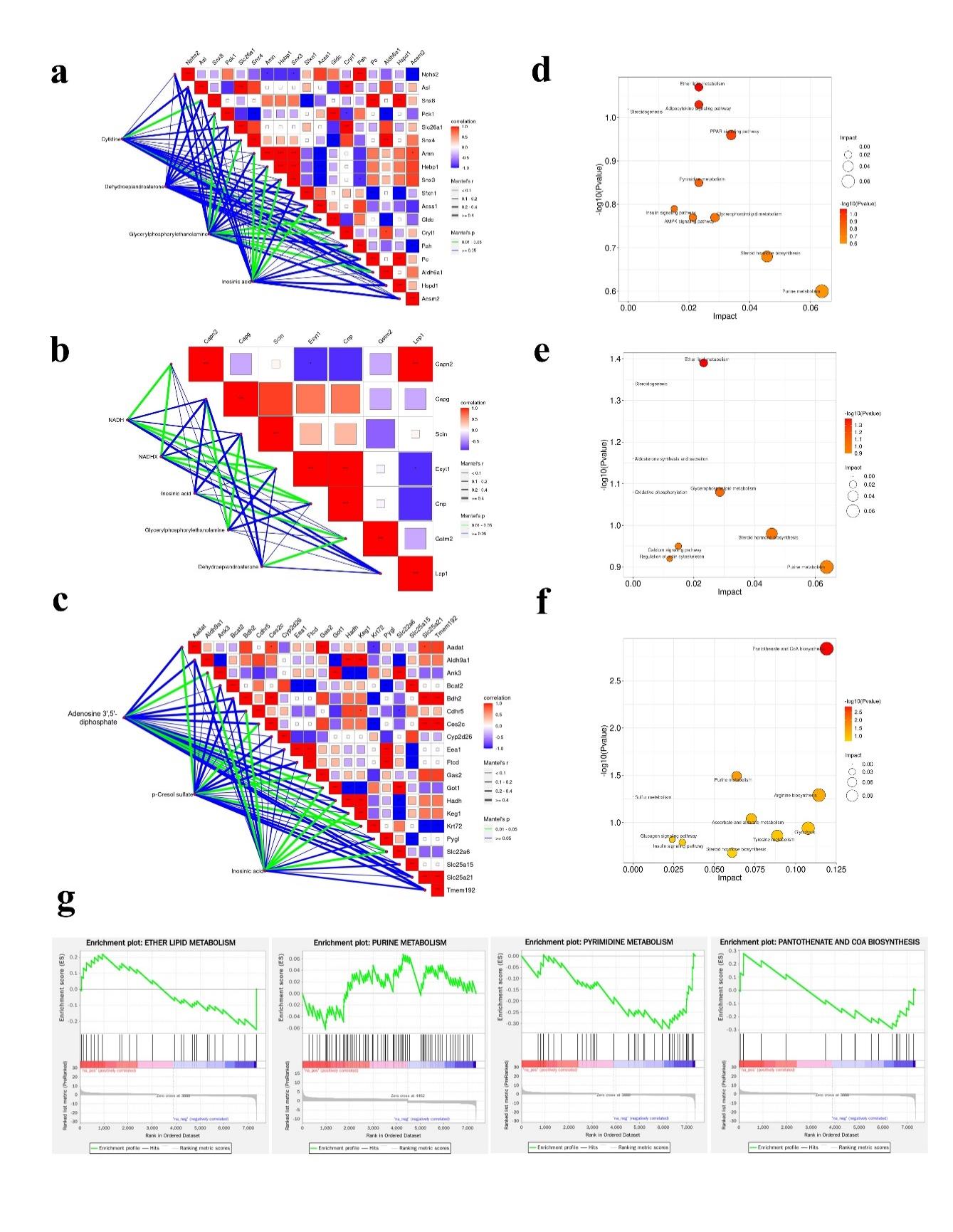


**Supplementary Figure S4** Pathway enrichment and correlation analyses between metabolite and protein alterations of different kidney regions. (a) Association of the metabolite levels and protein levels in Cor region-specific changes in response to MET treatment. (b) Correlations analysis of the metabolite and protein levels in the OM region in response to MET treatment. (c) Association of the metabolite levels and protein levels in IM region-specific changes in response to MET treatment. (d) Enriched pathway sets of metabolite and protein alterations in the Cor region related to MET response. (e) Pathway enrichment analysis of metabolite and protein alterations in the OM region related to MET response. (f) Enriched pathway sets of metabolite and protein alterations in the IM region related to MET response. (g) GSEA analysis of pathway enrichment.


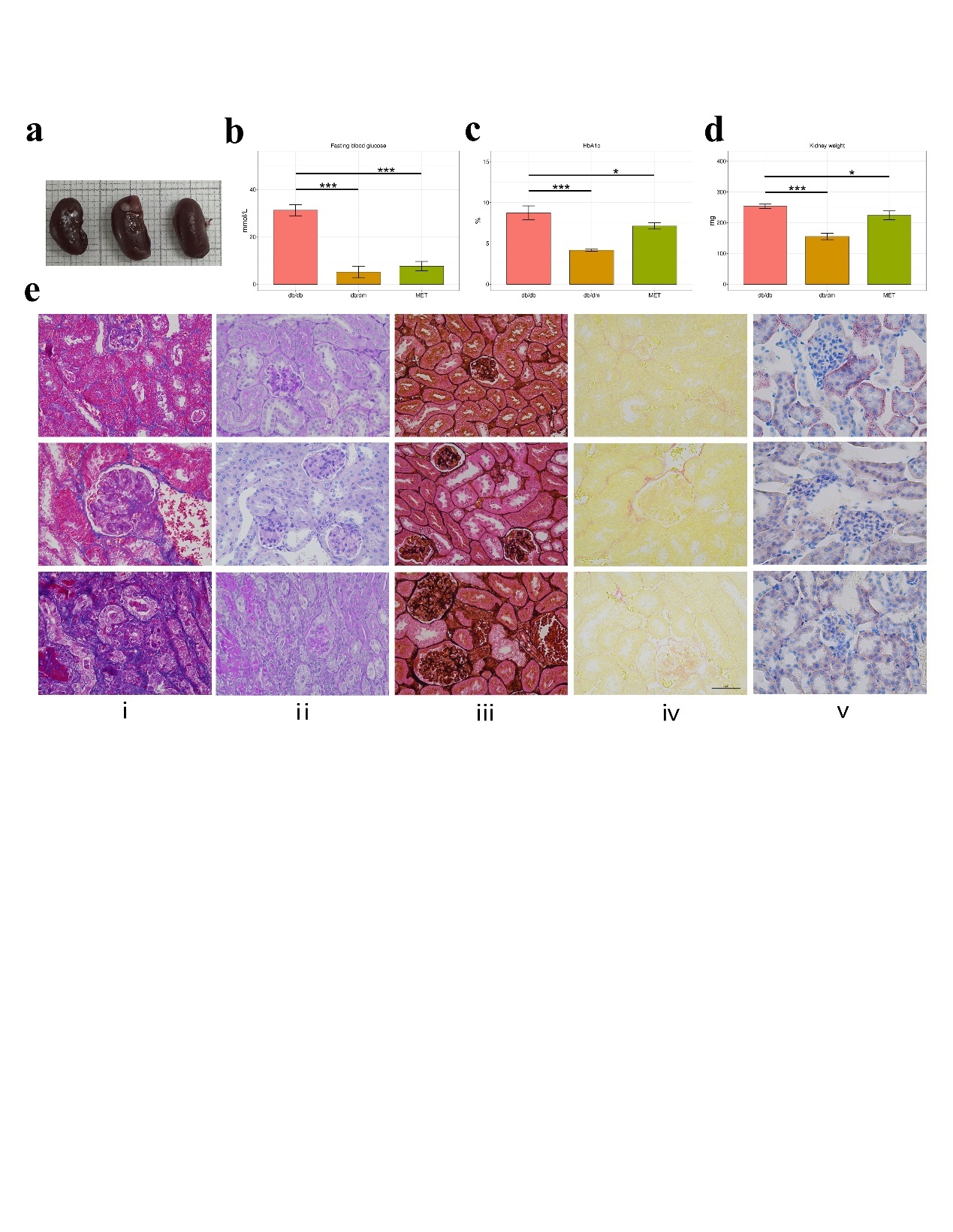


**Supplementary Figure S5** MET-ameliorated pathological and biochemical indicators in *db/db* mice. (a) Representative mouse kidneys of *db/dm* mice (left), *db/db* (middle) mice, and MET (right) group. (b) Quantitative analysis of blood glucose. (c) HOMA-IR analysis. (d) Quantitative analysis of kidney weight. (e) Masson staining (ⅰ), PAS staining (ⅱ), PASM staining (ⅲ), PSR staining (ⅳ), Oil Red staining (ⅴ) images (magnification 400×) of kidney tissue sections in *db/dm* mice (up), *db/db* mice (middle), and MET group (down).

**Supplementary Table S1** The metabolite biomarkers in *db/db* mouse kidney tissues selected from Z-score analysis.

| **Detected m/z** | **Ion** | **Adduct** | **Molecule Ids** | **Name** |
| --- | --- | --- | --- | --- |
| 187.0059 | C7H8O4S-H- | M-H | HMDB0011635 | p-cresol sulfate |
| 214.0469 | C5H14NO6P-H- | M-H | HMDB0000114 | Glycerylphosphorylethanolamine |
| 242.078 | C9H13N3O5-H- | M-H | HMDB0000089 | Cytidine |
| 287.0582 | C15H12O6-H- | M-H | HMDB0000077 | Dehydroepiandrosterone |
| 347.0373 | C10H13N4O8P-H- | M-H | HMDB0000175 | Inosinic acid |
| 426.0193 | C10H15N5O10P2-H- | M-H | HMDB0000061 | Adenosine 3’,5’-diphosphate |
| 664.1145 | C21H29N7O14P2-H- | M-H | HMDB0001487 | NADH |
| 682.1227 | C21H31N7O15P2-H- | M-H | HMDB0059644 | NADHX |

**Supplementary Table S2** The correlation analysis (Mantel test) matrix between metabolite expression levels and patient features in clinic.

| **Metabolites** | **Clinical parameters** | **Correlation** | ***P* value** | **Mantels_*r*** | **Mantels_*P*** |
| --- | --- | --- | --- | --- | --- |
| NADHX | eGFR | 0.46 | 0.00^*^ | ≥ 0.4 | < 0.01 |
| Cytidine | BUN | 0.24 | 0.01^*^ | 0.2−0.4 | < 0.01 |
| NADHX | Serum creatinine | 0.20 | 0.04^*^ | 0.2−0.4 | 0.01−0.05 |
| p-cresol sulfate | Serum creatinine | 0.24 | 0.05 | 0.2−0.4 | ≥ 0.05 |
| Adenosine 3’,5’-diphosphate | BUN | 0.34 | 0.06 | 0.2−0.4 | ≥ 0.05 |
| NADHX | Blood glucose | 0.29 | 0.06 | 0.2−0.4 | ≥ 0.05 |
| Dehydroepiandrosterone | Serum creatinine | 0.12 | 0.09 | 0.1−0.2 | ≥ 0.05 |
| Adenosine 3’,5’-diphosphate | Serum creatinine | 0.10 | 0.17 | 0.1−0.2 | ≥ 0.05 |
| p-cresol sulfate | eGFR | 0.02 | 0.28 | < 0.1 | ≥ 0.05 |
| Dehydroepiandrosterone | eGFR | 0.03 | 0.32 | < 0.1 | ≥ 0.05 |
| Dehydroepiandrosterone | BUN | 0.03 | 0.38 | < 0.1 | ≥ 0.05 |
| Cytidine | Serum creatinine | 0.00 | 0.40 | < 0.1 | ≥ 0.05 |
| NADHX | BUN | 0.00 | 0.43 | < 0.1 | ≥ 0.05 |
| p-cresol sulfate | BUN | −0.04 | 0.47 | < 0.1 | ≥ 0.05 |
| Dehydroepiandrosterone | Blood glucose | −0.02 | 0.49 | < 0.1 | ≥ 0.05 |
| Cytidine | Blood glucose | −0.04 | 0.61 | < 0.1 | ≥ 0.05 |
| Adenosine 3’,5’-diphosphate | Blood glucose | −0.06 | 0.62 | < 0.1 | ≥ 0.05 |
| Adenosine 3’,5’-diphosphate | eGFR | −0.09 | 0.70 | < 0.1 | ≥ 0.05 |
| p-cresol sulfate | Blood glucose | −0.14 | 0.81 | < 0.1 | ≥ 0.05 |
| Cytidine | eGFR | −0.07 | 0.86 | < 0.1 | ≥ 0.05 |

**Supplementary Table S3** The differentially expressed proteins in *db/db* mouse kidney Cor region.

| **No.** | **Gene name** | **Coverage [%]** | **Peptides** | **Unique peptides** | **KEGG KO No.** |
| --- | --- | --- | --- | --- | --- |
| 1 | *Acsm2* | 76.17 | 53 | 53 | K01896 |
| 2 | *Acss1* | 34.16 | 24 | 24 | K01895 |
| 3 | *Aldh6a1* | 49.35 | 30 | 30 | K00140 |
| 4 | *Amn* | 27.07 | 11 | 11 | K18259 |
| 5 | *Asl* | 49.78 | 26 | 26 | K01755 |
| 6 | *Cryl1* | 42.95 | 15 | 15 | K13247 |
| 7 | *Gldc* | 24.88 | 23 | 23 | K00281 |
| 8 | *Hebp1* | 36.84 | 7 | 7 |  |
| 9 | *Hspd1* | 54.8 | 42 | 42 | K04077 |
| 10 | *Nphs2* | 26.75 | 9 | 9 | K18268 |
| 11 | *Pah* | 56.07 | 28 | 28 | K00500 |
| 12 | *Pc* | 50.85 | 59 | 59 | K01958 |
| 13 | *Pck1* | 51.61 | 35 | 35 | K01596 |
| 14 | *Sfxn1* | 34.78 | 13 | 13 | K23500 |
| 15 | *Slc26a1* | 15.34 | 10 | 10 | K14700 |
| 16 | *Snx3* | 42.59 | 9 | 9 | K17918 |
| 17 | *Snx4* | 40 | 17 | 17 | K17919 |
| 18 | *Snx8* | 17.65 | 8 | 8 | K17922 |

**Supplementary Table S4** The differentially expressed proteins in *db/db* mouse kidney OM region.

| **No.** | **Gene name** | **Coverage [%]** | **Peptides** | **Unique peptides** | **KEGG KO No.** |
| --- | --- | --- | --- | --- | --- |
| 1 | *Capg* | 22.73 | 7 | 7 | K10368 |
| 2 | *Capn2* | 26.14 | 18 | 18 | K03853 |
| 3 | *Cnp* | 29.76 | 12 | 12 | K01121 |
| 4 | *Esyt1* | 25.37 | 28 | 28 |  |
| 5 | *Gstm2* | 71.56 | 19 | 16 | K00799 |
| 6 | *Lcp1* | 45.45 | 25 | 20 | K17276 |
| 7 | *Scin* | 40.14 | 28 | 28 | K05768 |

**Supplementary Table S5** The differentially expressed proteins in *db/db* mouse kidney IM region.

| **No.** | **Gene name** | **Coverage [%]** | **Peptides** | **Unique peptides** | **KEGG KO No.** |
| --- | --- | --- | --- | --- | --- |
| 1 | *Aadat* | 44.71 | 21 | 21 | K00825 |
| 2 | *Aldh9a1* | 50.4 | 29 | 29 | K00149 |
| 3 | *Ank3* | 33.35 | 59 | 51 | K10380 |
| 4 | *Bcat2* | 40.71 | 16 | 16 | K00826 |
| 5 | *Bdh2* | 46.94 | 14 | 14 | K25939 |
| 6 | *Cdhr5* | 10.23 | 8 | 8 | K16505 |
| 7 | *Ces2c* | 31.73 | 16 | 13 | K03927 |
| 8 | *Cyp2d26* | 38.4 | 20 | 20 | K07414 |
| 9 | *Eea1* | 50.11 | 71 | 70 | K12478 |
| 10 | *Ftcd* | 26.25 | 12 | 12 | K13990 |
| 11 | *Gas2* | 33.76 | 9 | 9 | K24627 |
| 12 | *Got1* | 63.44 | 27 | 27 | K14454 |
| 13 | *Hadh* | 40.76 | 18 | 18 | K00022 |
| 14 | *Keg1* | 50.17 | 17 | 17 | K00628 |
| 15 | *Krt72* | 3.65 | 2 | 1 | K07605 |
| 16 | *Pygl* | 35.18 | 33 | 33 | K00688 |
| 17 | *Slc22a6* | 12.11 | 8 | 8 | K08203 |
| 18 | *Slc25a15* | 32.23 | 10 | 10 | K15101 |
| 19 | *Slc25a21* | 39.26 | 13 | 13 | K15110 |
| 20 | *Tmem192* | 18.05 | 4 | 4 |  |

**Supplementary Table S6** Association of the metabolite expression and protein levels in the Cor region.

| Metabolite | Protein | Correlation | *P* value | Mantels_*r* | Mantels_*P* |
| --- | --- | --- | --- | --- | --- |
| Cytidine | Nphs2 | −0.87 | 0.04^*^ | < 0.1 | ≥ 0.05 |
| Cytidine | Asl | 0.84 | 0.10 | ≥ 0.4 | ≥ 0.05 |
| Cytidine | Sfxn1 | 0.83 | 0.07 | ≥ 0.4 | ≥ 0.05 |
| Cytidine | Hspd1 | 0.56 | 0.12 | ≥ 0.4 | ≥ 0.05 |
| Cytidine | Acss1 | 0.39 | 0.27 | 0.2−0.4 | ≥ 0.05 |
| Cytidine | Slc26a1 | 0.38 | 0.25 | 0.2−0.4 | ≥ 0.05 |
| Cytidine | Cryl1 | 0.22 | 0.28 | 0.2−0.4 | ≥ 0.05 |
| Cytidine | Gldc | 0.22 | 0.30 | 0.2−0.4 | ≥ 0.05 |
| Cytidine | Aldh6a1 | 0.21 | 0.30 | 0.2−0.4 | ≥ 0.05 |
| Cytidine | Snx4 | 0.15 | 0.30 | 0.1−0.2 | ≥ 0.05 |
| Cytidine | Pck1 | 0.05 | 0.42 | < 0.1 | ≥ 0.05 |
| Cytidine | Amn | 0.02 | 0.32 | < 0.1 | ≥ 0.05 |
| Cytidine | Snx3 | 0.00 | 0.35 | < 0.1 | ≥ 0.05 |
| Cytidine | Hebp1 | 0.00 | 0.35 | < 0.1 | ≥ 0.05 |
| Cytidine | Pah | −0.10 | 0.50 | < 0.1 | ≥ 0.05 |
| Cytidine | Acsm2 | −0.13 | 0.47 | < 0.1 | >= 0.05 |
| Dehydroepiandrosterone | Nphs2 | 0.87 | 0.02 | ≥ 0.4 | 0.01 - 0.05 |
| Dehydroepiandrosterone | Slc26a1 | 0.71 | 0.10 | ≥ 0.4 | ≥ 0.05 |
| Dehydroepiandrosterone | Snx8 | 0.65 | 0.12 | ≥ 0.4 | ≥ 0.05 |
| Dehydroepiandrosterone | Pc | 0.63 | 0.12 | ≥ 0.4 | ≥ 0.05 |
| Dehydroepiandrosterone | Cryl1 | 0.57 | 0.18 | ≥ 0.4 | ≥ 0.05 |
| Dehydroepiandrosterone | Gldc | 0.57 | 0.20 | ≥ 0.4 | ≥ 0.05 |
| Dehydroepiandrosterone | Aldh6a1 | 0.56 | 0.20 | ≥ 0.4 | ≥ 0.05 |
| Dehydroepiandrosterone | Sfxn1 | 0.56 | 0.12 | ≥ 0.4 | ≥ 0.05 |
| Dehydroepiandrosterone | Snx4 | 0.50 | 0.20 | ≥ 0.4 | ≥ 0.05 |
| Dehydroepiandrosterone | Pck1 | 0.37 | 0.23 | 0.2−0.4 | ≥ 0.05 |
| Dehydroepiandrosterone | Hspd1 | 0.29 | 0.12 | 0.2−0.4 | ≥ 0.05 |
| Dehydroepiandrosterone | Acss1 | 0.16 | 0.42 | 0.1−0.2 | ≥ 0.05 |
| Dehydroepiandrosterone | Pah | 0.06 | 0.50 | < 0.1 | ≥ 0.05 |
| Dehydroepiandrosterone | Amn | −0.09 | 0.42 | < 0.1 | ≥ 0.05 |
| Dehydroepiandrosterone | Snx3 | −0.10 | 0.42 | < 0.1 | ≥ 0.05 |
| Dehydroepiandrosterone | Hebp1 | −0.10 | 0.42 | < 0.1 | ≥ 0.05 |
| Dehydroepiandrosterone | Acsm2 | −0.16 | 0.43 | < 0.1 | ≥ 0.05 |
| Glycerylphosphorylethanolamine | Nphs2 | 0.91 | 0.03 | ≥ 0.4 | 0.01−0.05 |
| Glycerylphosphorylethanolamine | Pah | 0.92 | 0.10 | ≥ 0.4 | ≥ 0.05 |
| Glycerylphosphorylethanolamine | Aldh6a1 | 0.76 | 0.02 | ≥ 0.4 | 0.01−0.05 |
| Glycerylphosphorylethanolamine | Gldc | 0.75 | 0.02 | ≥ 0.4 | 0.01−0.05 |
| Glycerylphosphorylethanolamine | Cryl1 | 0.74 | 0.03 | ≥ 0.4 | 0.01−0.05 |
| Glycerylphosphorylethanolamine | Slc26a1 | 0.69 | 0.10 | ≥ 0.4 | ≥ 0.05 |
| Glycerylphosphorylethanolamine | Acsm2 | 0.59 | 0.15 | ≥ 0.4 | ≥ 0.05 |
| Glycerylphosphorylethanolamine | Hebp1 | 0.47 | 0.30 | ≥ 0.4 | ≥ 0.05 |
| Glycerylphosphorylethanolamine | Snx3 | 0.47 | 0.30 | ≥ 0.4 | ≥ 0.05 |
| Glycerylphosphorylethanolamine | Amn | 0.46 | 0.30 | ≥ 0.4 | ≥ 0.05 |
| Glycerylphosphorylethanolamine | Asl | 0.28 | 0.12 | 0.2−0.4 | ≥ 0.05 |
| Glycerylphosphorylethanolamine | Acss1 | 0.22 | 0.27 | 0.2−0.4 | ≥ 0.05 |
| Glycerylphosphorylethanolamine | Hspd1 | 0.10 | 0.57 | < 0.1 | ≥ 0.05 |
| Glycerylphosphorylethanolamine | Sfxn1 | −0.06 | 0.50 | < 0.1 | ≥ 0.05 |
| Glycerylphosphorylethanolamine | Pc | −0.10 | 0.43 | < 0.1 | ≥ 0.05 |
| Glycerylphosphorylethanolamine | Snx8 | −0.12 | 0.43 | < 0.1 | ≥ 0.05 |
| Inosinic acid | Nphs2 | 0.89 | 0.03 | ≥ 0.4 | 0.01−0.05 |
| Inosinic acid | Amn | 0.83 | 0.02 | ≥ 0.4 | 0.01−0.05 |
| Inosinic acid | Sfxn1 | 0.83 | 0.07 | ≥ 0.4 | ≥ 0.05 |
| Inosinic acid | Snx3 | 0.82 | 0.02 | ≥ 0.4 | 0.01−0.05 |
| Inosinic acid | Hebp1 | 0.82 | 0.02 | ≥ 0.4 | 0.01−0.05 |
| Inosinic acid | Pc | 0.74 | 0.07 | ≥ 0.4 | ≥ 0.05 |
| Inosinic acid | Snx8 | 0.70 | 0.07 | ≥ 0.4 | ≥ 0.05 |
| Inosinic acid | Acsm2 | 0.69 | 0.07 | ≥ 0.4 | ≥ 0.05 |
| Inosinic acid | Pah | 0.40 | 0.12 | 0.2−0.4 | ≥ 0.05 |
| Inosinic acid | Asl | 0.23 | 0.18 | 0.2−0.4 | ≥ 0.05 |
| Inosinic acid | Pck1 | 0.06 | 0.43 | < 0.1 | ≥ 0.05 |
| Inosinic acid | Snx4 | −0.06 | 0.43 | < 0.1 | ≥ 0.05 |
| Inosinic acid | Slc26a1 | −0.07 | 0.42 | < 0.1 | ≥ 0.05 |
| Inosinic acid | Aldh6a1 | −0.14 | 0.43 | < 0.1 | ≥ 0.05 |
| Inosinic acid | Cryl1 | −0.15 | 0.42 | < 0.1 | ≥ 0.05 |
| Inosinic acid | Gldc | −0.15 | 0.43 | < 0.1 | ≥ 0.05 |

**Supplementary Table S7** Association of the metabolite levels and protein levels in the OM region.

| Metabolite | Protein | Correlation | *P* value | Mantels_*r* | Mantels_*P* |
| --- | --- | --- | --- | --- | --- |
| Adenosine 3',5'-diphosphate | Aldh9a1 | 0.99 | 0.07 | ≥ 0.4 | ≥ 0.05 |
| Inosinic acid | Slc22a6 | 0.98 | 0.03^*^ | ≥ 0.4 | 0.01−0.05 |
| Adenosine 3',5'-diphosphate | Bdh2 | 0.98 | 0.07 | ≥ 0.4 | ≥ 0.05 |
| Inosinic acid | Cdhr5 | 0.96 | 0.02^*^ | ≥ 0.4 | 0.01−0.05 |
| Inosinic acid | Ank3 | 0.95 | 0.03^*^ | ≥ 0.4 | 0.01−0.05 |
| Inosinic acid | Keg1 | 0.95 | 0.03^*^ | ≥ 0.4 | 0.01−0.05 |
| Inosinic acid | Hadh | 0.94 | 0.03^*^ | ≥ 0.4 | 0.01−0.05 |
| p-Cresol sulfate | Cyp2d26 | 0.92 | 0.10 | ≥ 0.4 | ≥ 0.05 |
| Adenosine 3',5'-diphosphate | Tmem192 | 0.92 | 0.07 | ≥ 0.4 | ≥ 0.05 |
| Adenosine 3',5'-diphosphate | Got1 | 0.91 | 0.05 | ≥ 0.4 | 0.01−0.05 |
| Inosinic acid | Got1 | 0.88 | 0.03^*^ | ≥ 0.4 | 0.01−0.05 |
| p-Cresol sulfate | Gas2 | 0.87 | 0.02^*^ | ≥ 0.4 | 0.01−0.05 |
| p-Cresol sulfate | Aadat | 0.85 | 0.02^*^ | ≥ 0.4 | 0.01−0.05 |
| Adenosine 3',5'-diphosphate | Hadh | 0.82 | 0.07 | ≥ 0.4 | ≥ 0.05 |
| Adenosine 3',5'-diphosphate | Ank3 | 0.81 | 0.05 | ≥ 0.4 | 0.01−0.05 |
| Adenosine 3',5'-diphosphate | Keg1 | 0.80 | 0.07 | ≥ 0.4 | ≥ 0.05 |
| p-Cresol sulfate | Ces2c | 0.80 | 0.03^*^ | ≥ 0.4 | 0.01−0.05 |
| Adenosine 3',5'-diphosphate | Slc25a21 | 0.79 | 0.07 | ≥ 0.4 | ≥ 0.05 |
| p-Cresol sulfate | Krt72 | 0.77 | 0.03^*^ | ≥ 0.4 | 0.01−0.05 |
| Adenosine 3',5'-diphosphate | Slc22a6 | 0.73 | 0.05 | ≥ 0.4 | 0.01−0.05 |
| p-Cresol sulfate | Slc25a21 | 0.73 | 0.07 | ≥ 0.4 | ≥ 0.05 |
| Adenosine 3',5'-diphosphate | Krt72 | 0.68 | 0.05 | ≥ 0.4 | 0.01−0.05 |
| Inosinic acid | Aldh9a1 | 0.68 | 0.07 | ≥ 0.4 | ≥ 0.05 |
| Adenosine 3',5'-diphosphate | Ces2c | 0.64 | 0.12 | ≥ 0.4 | ≥ 0.05 |
| p-Cresol sulfate | Tmem192 | 0.61 | 0.12 | ≥ 0.4 | ≥ 0.05 |
| Adenosine 3',5'-diphosphate | Aadat | 0.60 | 0.17 | ≥ 0.4 | ≥ 0.05 |
| Adenosine 3',5'-diphosphate | Gas2 | 0.59 | 0.17 | ≥ 0.4 | ≥ 0.05 |
| Adenosine 3',5'-diphosphate | Cdhr5 | 0.50 | 0.12 | ≥ 0.4 | ≥ 0.05 |
| p-Cresol sulfate | Bdh2 | 0.47 | 0.12 | ≥ 0.4 | ≥ 0.05 |
| Inosinic acid | Bdh2 | 0.47 | 0.30^*^ | ≥ 0.4 | ≥ 0.05 |
| p-Cresol sulfate | Ftcd | 0.45 | 0.30 | ≥ 0.4 | ≥ 0.05 |
| p-Cresol sulfate | Eea1 | 0.42 | 0.30 | ≥ 0.4 | ≥ 0.05 |
| p-Cresol sulfate | Bcat2 | 0.42 | 0.30 | ≥ 0.4 | ≥ 0.05 |
| Inosinic acid | Slc25a15 | 0.40 | 0.25 | 0.2−0.4 | ≥ 0.05 |
| Inosinic acid | Pygl | 0.34 | 0.25 | 0.2−0.4 | ≥ 0.05 |
| p-Cresol sulfate | Pygl | 0.31 | 0.27 | 0.2−0.4 | ≥ 0.05 |
| Inosinic acid | Tmem192 | 0.30 | 0.30 | 0.2−0.4 | ≥ 0.05 |
| p-Cresol sulfate | Aldh9a1 | 0.28 | 0.27 | 0.2−0.4 | ≥ 0.05 |
| p-Cresol sulfate | Slc25a15 | 0.28 | 0.27 | 0.2−0.4 | ≥ 0.05 |
| Inosinic acid | Bcat2 | 0.11 | 0.25 | 0.1−0.2 | ≥ 0.05 |
| Inosinic acid | Eea1 | 0.10 | 0.25 | 0.1−0.2 | ≥ 0.05 |
| Adenosine 3',5'-diphosphate | Cyp2d26 | 0.09 | 0.50 | < 0.1 | ≥ 0.05 |
| Inosinic acid | Slc25a21 | 0.09 | 0.30 | < 0.1 | ≥ 0.05 |
| p-Cresol sulfate | Got1 | 0.08 | 0.42 | < 0.1 | ≥ 0.05 |
| Inosinic acid | Ftcd | 0.03 | 0.28 | < 0.1 | ≥ 0.05 |
| Adenosine 3',5'-diphosphate | Slc25a15 | 0.01 | 0.42 | < 0.1 | ≥ 0.05 |
| p-Cresol sulfate | Hadh | −0.01 | 0.42 | < 0.1 | ≥ 0.05 |
| Adenosine 3',5'-diphosphate | Pygl | −0.01 | 0.42 | < 0.1 | ≥ 0.05 |
| p-Cresol sulfate | Keg1 | −0.02 | 0.42 | < 0.1 | ≥ 0.05 |
| p-Cresol sulfate | Ank3 | −0.03 | 0.42 | < 0.1 | ≥ 0.05 |
| Inosinic acid | Krt72 | −0.03 | 0.35 | < 0.1 | ≥ 0.05 |
| p-Cresol sulfate | Cdhr5 | −0.08 | 0.57 | < 0.1 | ≥ 0.05 |
| Inosinic acid | Ces2c | −0.10 | 0.38 | < 0.1 | ≥ 0.05 |
| Inosinic acid | Aadat | −0.12 | 0.40 | < 0.1 | ≥ 0.05 |
| Inosinic acid | Gas2 | −0.12 | 0.43 | < 0.1 | ≥ 0.05 |
| Adenosine 3',5'-diphosphate | Eea1 | −0.13 | 0.42 | < 0.1 | ≥ 0.05 |
| Adenosine 3',5'-diphosphate | Bcat2 | −0.14 | 0.42 | < 0.1 | ≥ 0.05 |
| Adenosine 3',5'-diphosphate | Ftcd | −0.15 | 0.42 | < 0.1 | ≥ 0.05 |
| p-Cresol sulfate | Slc22a6 | −0.16 | 0.42 | < 0.1 | ≥ 0.05 |
| Inosinic acid | Cyp2d26 | −0.17 | 0.42 | < 0.1 | ≥ 0.05 |

**Supplementary Table S8** Association of the metabolite levels and protein levels in the IM region.

| Metabolite | Protein | Correlation | *P* value | Mantels_*r* | Mantels_*P* |
| --- | --- | --- | --- | --- | --- |
| Glycerylphosphorylethanolamine | Capg | 0.99 | 0.02^*^ | ≥ 0.4 | 0.01−0.05 |
| NADHX | Cnp | 0.98 | 0.02^*^ | ≥ 0.4 | 0.01−0.05 |
| NADHX | Esyt1 | 0.96 | 0.02^*^ | ≥ 0.4 | 0.01−0.05 |
| Glycerylphosphorylethanolamine | Scin | 0.94 | 0.03^*^ | ≥ 0.4 | 0.01−0.05 |
| NADH | Capn2 | 0.91 | 0.03^*^ | ≥ 0.4 | 0.01−0.05 |
| NADH | Cnp | 0.87 | 0.02^*^ | ≥ 0.4 | 0.01−0.05 |
| Inosinic acid | Gstm2 | 0.86 | 0.02^*^ | ≥ 0.4 | 0.01−0.05 |
| NADH | Esyt1 | 0.79 | 0.02^*^ | ≥ 0.4 | 0.01−0.05 |
| NADHX | Capn2 | 0.78 | 0.03^*^ | ≥ 0.4 | 0.01−0.05 |
| Dehydroepiandrosterone | Scin | 0.77 | 0.07 | ≥ 0.4 | ≥ 0.05 |
| Dehydroepiandrosterone | Gstm2 | 0.76 | 0.02^*^ | ≥ 0.4 | 0.01−0.05 |
| Glycerylphosphorylethanolamine | Esyt1 | 0.70 | 0.10 | ≥ 0.4 | ≥ 0.05 |
| Inosinic acid | Scin | 0.63 | 0.15 | ≥ 0.4 | ≥ 0.05 |
| NADH | Lcp1 | 0.62 | 0.12 | ≥ 0.4 | ≥ 0.05 |
| Dehydroepiandrosterone | Capg | 0.58 | 0.17 | ≥ 0.4 | ≥ 0.05 |
| Glycerylphosphorylethanolamine | Cnp | 0.57 | 0.25 | ≥ 0.4 | ≥ 0.05 |
| NADHX | Capg | 0.54 | 0.15 | ≥ 0.4 | ≥ 0.05 |
| NADHX | Lcp1 | 0.48 | 0.12 | ≥ 0.4 | ≥ 0.05 |
| Inosinic acid | Capg | 0.40 | 0.30 | ≥ 0.4 | ≥ 0.05 |
| NADHX | Scin | 0.40 | 0.30 | 0.2−0.4 | ≥ 0.05 |
| NADH | Capg | 0.30 | 0.28 | 0.2−0.4 | ≥ 0.05 |
| Inosinic acid | Lcp1 | 0.23 | 0.27 | 0.2−0.4 | ≥ 0.05 |
| NADH | Scin | 0.20 | 0.30 | 0.1−0.2 | ≥ 0.05 |
| Dehydroepiandrosterone | Esyt1 | 0.16 | 0.42 | 0.1−0.2 | ≥ 0.05 |
| Dehydroepiandrosterone | Lcp1 | 0.10 | 0.43 | 0.1−0.2 | ≥ 0.05 |
| Dehydroepiandrosterone | Cnp | 0.06 | 0.42 | < 0.1 | ≥ 0.05 |
| Glycerylphosphorylethanolamine | Capn2 | 0.05 | 0.30 | < 0.1 | ≥ 0.05 |
| Glycerylphosphorylethanolamine | Gstm2 | 0.03 | 0.35 | < 0.1 | ≥ 0.05 |
| Inosinic acid | Esyt1 | −0.02 | 0.57 | < 0.1 | ≥ 0.05 |
| NADH | Gstm2 | −0.04 | 0.47 | < 0.1 | ≥ 0.05 |
| Inosinic acid | Cnp | −0.08 | 0.57 | < 0.1 | ≥ 0.05 |
| Glycerylphosphorylethanolamine | Lcp1 | −0.09 | 0.42 | < 0.1 | ≥ 0.05 |
| NADHX | Gstm2 | −0.10 | 0.43 | < 0.1 | ≥ 0.05 |
| Inosinic acid | Capn2 | −0.12 | 0.42 | < 0.1 | ≥ 0.05 |
| Dehydroepiandrosterone | Capn2 | −0.13 | 0.43 | < 0.1 | ≥ 0.05 |

**Supplementary Table S9** KEGG enrichment analysis for metabolites and proteins in the Cor region.

| Pathway_name | Hits | *P* value | Impact |
| --- | --- | --- | --- |
| Purine metabolism | 1 | 0.25181 | 0.063725 |
| Steroid hormone biosynthesis | 1 | 0.20996 | 0.045685 |
| PPAR signaling pathway | 1 | 0.10851 | 0.033898 |
| Glycerophospholipid metabolism | 1 | 0.17012 | 0.028571 |
| Ether lipid metabolism | 1 | 0.085905 | 0.023256 |
| Adipocytokine signaling pathway | 1 | 0.092743 | 0.023256 |
| Pyrimidine metabolism | 1 | 0.14253 | 0.023256 |
| AMPK signaling pathway | 1 | 0.17012 | 0.021277 |
| Insulin signaling pathway | 1 | 0.16383 | 0.015152 |
| Steroidogenesis | 1 | 0.096145 | 0 |

**Supplementary Table S10** KEGG enrichment analysis for metabolites and proteins in the OM region.

| Pathway_name | Hits | *P* value | Impact |
| --- | --- | --- | --- |
| Purine metabolism | 1 | 0.12658 | 0.063725 |
| Steroid hormone biosynthesis | 1 | 0.10412 | 0.045685 |
| Glycerophospholipid metabolism | 1 | 0.083317 | 0.028571 |
| Ether lipid metabolism | 1 | 0.041037 | 0.023256 |
| Calcium signaling pathway | 1 | 0.11148 | 0.014925 |
| Regulation of actin cytoskeleton | 1 | 0.1214 | 0.012048 |
| Steroidogenesis | 1 | 0.046063 | 0 |
| Aldosterone synthesis and secretion | 1 | 0.069223 | 0 |
| Oxidative phosphorylation | 1 | 0.083317 | 0 |

**Supplementary Table S11** KEGG enrichment analysis for metabolites and proteins in the IM region.

| Pathway_name | Hits | *P* value | Impact |
| --- | --- | --- | --- |
| Pantothenate and CoA biosynthesis | 2 | 0.001446 | 0.11905 |
| Arginine biosynthesis | 1 | 0.050984 | 0.11429 |
| Glycolysis | 1 | 0.11409 | 0.10769 |
| Tyrosine metabolism | 1 | 0.13713 | 0.08871 |
| Ascorbate and aldarate metabolism | 1 | 0.090469 | 0.072727 |
| Purine metabolism | 2 | 0.03252 | 0.063725 |
| Steroid hormone biosynthesis | 1 | 0.20996 | 0.060914 |
| Insulin signaling pathway | 1 | 0.16383 | 0.030303 |
| Glucagon signaling pathway | 1 | 0.15004 | 0.024096 |
| Sulfur metabolism | 1 | 0.053351 | 0 |

**Supplementary Table S12** The clinical characteristics of all the subjects.

|  | DN | HC | *P*-value |
| --- | --- | --- | --- |
| Number | 17 | 17 | - |
| Male/female | 4/13 | 3/14 | - |
| Age | 57.94 ± 7.90 | 61.94 ± 5.99 | 0.11 |
| BMI (kg/m^2^) | 23.87 ± 2.49 | 24.38 ± 2.05 | 0.52 |
| Serum creatinine (μmol/L) | 508.15 ± 261.80 | 75.61 ± 13.18 | 0.00 |
| BUN (mmol/L) | 19.14 ± 8.54 | 4.90 ± 1.48 | 0.00 |
| eGFR (mL/(min × 1.73 m^2^)) | 15.06 ± 13.80 | 95.59 ± 12.97 | 0.00 |
| Blood uric acid (μmol/L) | 435.76 ± 135.31 | 371.88 ± 73.86 | 0.10 |
| Fasting blood glucose (mmol/L) | 9.44 ± 2.61 | 5.19 ± 0.54 | 0.00 |
| TC (mmol/L) | 4.73 ± 0.98 | 5.81 ± 1.36 | 0.01 |
| TG (mmol/L) | 1.81 ± 1.32 | 1.80 ± 0.83 | 1.00 |
| HDL (mmol/L) | 1.01 ± 0.35 | 1.20 ± 0.25 | 0.08 |
| LDL (mmol/L) | 2.46 ± 0.69 | 3.25 ± 0.99 | 0.01 |

Note: BUN, blood urea nitrogen; eGFR: estimated glomerular filtration rate.
